# A yeast chemogenomic screen identifies pathways that modulate adipic acid toxicity

**DOI:** 10.1016/j.isci.2021.102327

**Published:** 2021-03-18

**Authors:** Eugene Fletcher, Kevin Mercurio, Elizabeth A. Walden, Kristin Baetz

**Affiliations:** 1 Ottawa Institute of Systems Biology, Department of Biochemistry, Microbiology and Immunology, University of Ottawa, 451 Smyth Road, Ottawa, ON K1H 8M5, Canada

**Keywords:** Cell Biology, Systems Biology

## Abstract

Adipic acid production by yeast fermentation is gaining attention as a renewable source of platform chemicals for making nylon products. However, adipic acid toxicity inhibits yeast growth and fermentation. Here, we performed a chemogenomic screen in *Saccharomyces cerevisiae* to understand the cellular basis of adipic acid toxicity. Our screen revealed that *KGD1* (a key gene in the tricarboxylic acid cycle) deletion improved tolerance to adipic acid and its toxic precursor, catechol. Conversely, disrupting ergosterol biosynthesis as well as protein trafficking and vacuolar transport resulted in adipic acid hypersensitivity. Notably, we show that adipic acid disrupts the Membrane Compartment of Can1 (MCC) on the plasma membrane and impacts endocytosis. This was evidenced by the rapid internalization of Can1 for vacuolar degradation. As ergosterol is an essential component of the MCC and protein trafficking mechanisms are required for endocytosis, we highlight the importance of these cellular processes in modulating adipic acid toxicity.

## Highlights

• Deletion of the TCA cycle gene *KGD1* improves tolerance to adipic acid and catechol
• Ergosterol and Pdr12 play non-overlapping roles protecting cell from adipic acid
• Adipic acid-induced plasma membrane localization of Pdr12 is independent of ergosterol
• Adipic acid disrupts the Membrane Compartment of Can1 (MCC) and induces endocytosis

## Graphical abstract

**Figure:**
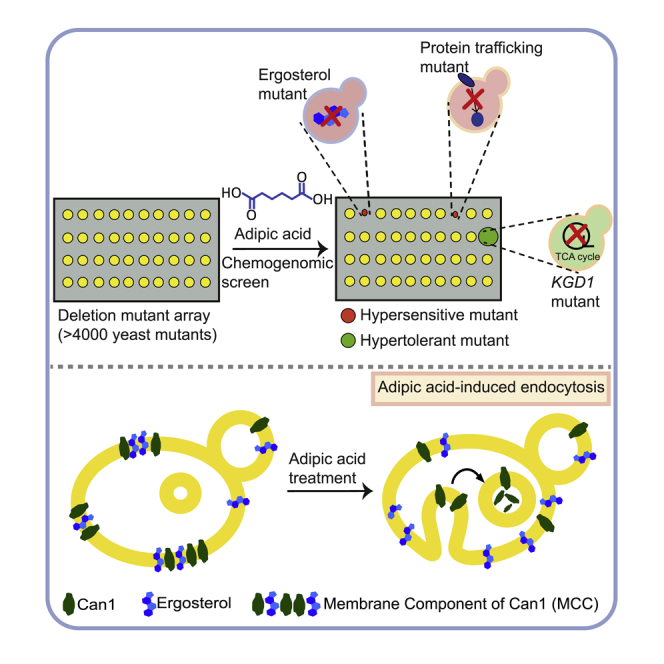

## Introduction

Adipic acid has been described as the most important dicarboxylic acid as it is a valuable platform chemical for the production of nylon-6,6 (Polen et al., 2013). The global need for adipic acid has been projected to increase because it can be converted to other products including plasticizers, plastics, lubricants, and a food ingredient in gelatin (Musser, 2000; Polen et al., 2013; Weber et al., 2012). Nearly all of the world's adipic acid is currently produced from a chemical process that involves the conversion of benzene to adipic acid through several chemical reactions requiring an oxidation reaction of nitric acid and air (Musser, 2000). These chemicals are costly and toxic to the environment and the nitric acid oxidation step results in the production of nitrous oxide gas, a greenhouse gas contributing to global warming and thinning of the ozone layer. Indeed, it is estimated that about 10% of the worldwide nitrous oxide gas released yearly into the atmosphere comes from adipic acid production (Alini et al., 2007).

Considering these challenges in the chemical synthesis of adipic acid, there has been a huge drive toward a more sustainable and environment friendly production of adipic acid by microbial fermentation. In this bioprocess, microorganisms such as bacteria and yeast are engineered to convert glucose to adipic acid. Yeasts, particularly *Saccharomyces cerevisiae*, are a preferred microbial host for adipic acid fermentation via the shikimate pathway, primarily because yeast metabolism has been extensively characterized and is easy to engineer (Averesch and Krömer, 2018; Nevoigt, 2008). Also, yeasts have previously been shown to be more tolerant to adipic acid toxicity than bacteria (Karlsson et al., 2017) and *S*. *cerevisiae* is generally robust to industrial stresses including low pH conditions (Chen et al., 2009; Fletcher et al., 2017), making it suitable for the production of organic acids including adipic acid. Although progress has steadily been made to produce adipic acid using engineered yeasts (Raj et al., 2018), the process is constrained by the toxicity of adipic acid to yeast. Hence, product toxicity is a key factor that should be considered during the design of engineered strains as this will greatly impact the final titers and productivity of the strain during the industrial production of adipic acid on a commercial scale.

Currently, the exact mechanism of adipic acid toxicity is unclear but is likely similar to the generic mechanism for organic acid toxicity. At a pH lower than the pKa of the acid (adipic acid pKa1 = 4.41, pKa2 = 5.41), organic acids freely diffuse into the cell where they then dissociate in the cytosol at a pH ∼7.0 into the anionic form, which is unable to diffuse back out of the cell due to the hydrophilic nature of the anions (Jarboe et al., 2013). In order to prevent accumulation and toxicity of the anionic form of the acid, the cell exports the anion using specific transmembrane pumps. These transmembrane pumps have broad specificities to groups of organic acids. For example, the yeast ATP-binding cassette (ABC) superfamily transporter, Pdr12, has been reported to pump propionate, benzoate, and sorbate out of the cell but not octanoate (Kren et al., 2003; Piper et al., 1998). The transporter Qdr3, belonging to the major facilitator superfamily class, was identified as playing a role in muconic acid production and tolerance to three dicarboxylic acids including adipic acid (Pereira et al., 2019). Even though the study did not show efflux of adipate by Qdr3, it demonstrated that overexpressing Qdr3 resulted in yeast tolerance to adipic acid.

Although the effect of low pH in yeast has been well established (Piper et al., 2001; Ullah et al., 2013) the actual toxicity of organic acids, which stems from the rapid intracellular accumulation of the anion at low pH, also results in specific cellular responses. Currently, knowledge of these cellular responses to dicarboxylic acids such as adipic acid remains unclear. However, the biological mechanisms underlying the toxicity of monocarboxylic acids including sorbic acid, benzoic acid, propionic acid, and acetic acid have been thoroughly investigated (Mira et al., 2010b). Transcriptomic analyses for monocarboxylic acids (acetic acid, propionic acid, benzoic acid, and sorbic acid) revealed similar transcriptional responses (Abbott et al., 2008) and also showed that four major transcriptional pathways regulated by Haa1, War1, Rim101, and Msn2/Msn4 modulate organic acid toxicity. This was observed across the different monocarboxylic acids. Functional genomics studies, on the other hand, indicated very specific toxicity mechanisms for each acid. For instance, genes associated with carbohydrate metabolism and ribosome biogenesis were specific to acetic acid, whereas genes involved in cell wall protein synthesis, tryptophan biosynthesis, and respiration were specific to benzoic acid, sorbic acid, and propionic acid, respectively (Kren et al., 2003; Mira et al., 2009, 2010b; Schüller et al., 2004). Furthermore, Trk1 involved in potassium influx was associated with propionic acid response (Xu et al., 2019). Owing to the very specific nature of the genetic mechanisms associated with different monocarboxylic acids it was not surprising that common genes were not identified for yeast evolved in parallel to three different dicarboxylic acids (glutaric acid, adipic acid, and pimelic acid) (Pereira et al., 2019). Therefore, monocarboxylic acid tolerance mechanisms in yeast cannot be used as an effective predictor of dicarboxylic acid tolerance. Toward improving adipic acid tolerance in *S*. *cerevisiae*, it is crucial to identify specific mechanisms underlying its toxicity and key genetic targets that can be engineered for tolerance.

Chemogenomic screens have previously been used to identify mechanisms of toxicity not only for organic acids (Hazelwood et al., 2010; Kawahata et al., 2006; Mira et al., 2009; Piotrowski et al., 2015; Schüller et al., 2004) but also for several other toxic compounds including fermentation inhibitors (Fletcher et al., 2019; Skerker et al., 2013). This screen allows the identification of hypersensitive mutants that show significant growth defects in the presence of a toxic compound. It also allows the identification of hypertolerant mutants lacking a suppressor gene that when deleted results in improved growth in the presence of the inhibitor compound. Despite the promising potential of adipic acid production by yeast fermentation, no previous study has reported specific mechanisms underlying the acid's toxicity to yeast. Here, we utilized a yeast chemogenomic screen to identify unique biological processes that modulate adipic acid toxicity, which can be potentially targeted to engineer tolerance in yeast.

## Results

### Chemogenomic screen reveals a role for ergosterol and protein trafficking in adipic acid tolerance

In order to identify biological processes underlying adipic acid toxicity in yeast, we performed a chemogenomic screen using the *S*. *cerevisiae* haploid deletion mutant array (DMA) collection to identify mutants that are hypersensitive or have increased tolerance to a sublethal concentration (80 mM) of adipic acid. The yeast DMA collection was pinned onto YPD agar plates containing 80 mM adipic acid (dissolved in DMSO), which lowered the pH of the agar plates to 4.0. To specifically identify mutants with modulated growth on adipic acid and not solely decreased pH, as a control the DMA collection was also pinned onto YPD plates containing DMSO (solvent control) and buffered at pH 4.0. We incubated the plates for 48 h after which the colony sizes of mutants grown on the adipic acid plates were compared with those of the pH 4.0 control plates and were analyzed using the synthetic genetic array (SGA) tools (Wagih et al., 2013). We identified a total of 77 adipic acid-sensitive mutants (SGA cutoff < −0.3) and one mutant that conferred improved growth on adipic acid (Table S1). To eliminate any false-positives, we quantified the growth rate of the 77 mutants in YPD medium supplemented with adipic at pH 4.0. The ratio of growth rate in the presence of adipic acid to that in the absence of adipic acid was obtained as the PRECOG score (Fernandez-Ricaud et al., 2016), which represented the level of sensitivity or tolerance of the mutants to adipic acid. Using a PRECOG score of ≥2, signifying more than 50% growth inhibition in the presence of adipic acid, we confirmed 43 mutants to be sensitive to adipic acid. In contrast only one suppressor or deletion mutant was confirmed to grow better in adipic acid (PRECOG score ≤0.8).

To further validate that our screen specifically selected mutants sensitive to adipic acid and not to the low pH (pH 4.0) conferred by adipic acid, we compared our data with a previously reported chemogenomic screen for pH 4.0 toxicity (Shin et al., 2016). We found no overlap between our data and the 129 genes identified in the low pH screen (Shin et al., 2016) (Table S1), giving a strong indication that the sensitive strains obtained in our screen have been selected for specific sensitivity to adipic acid. Previous work had reported that overexpression of *PDR12* improved tolerance to adipic acid when overexpressed (Pereira et al., 2019), and our screen identified *pdr12Δ* cells as being hypersensitive to adipic acid, providing further evidence that the chemogenomic screen is specific to adipic acid.

**Figure 1:**
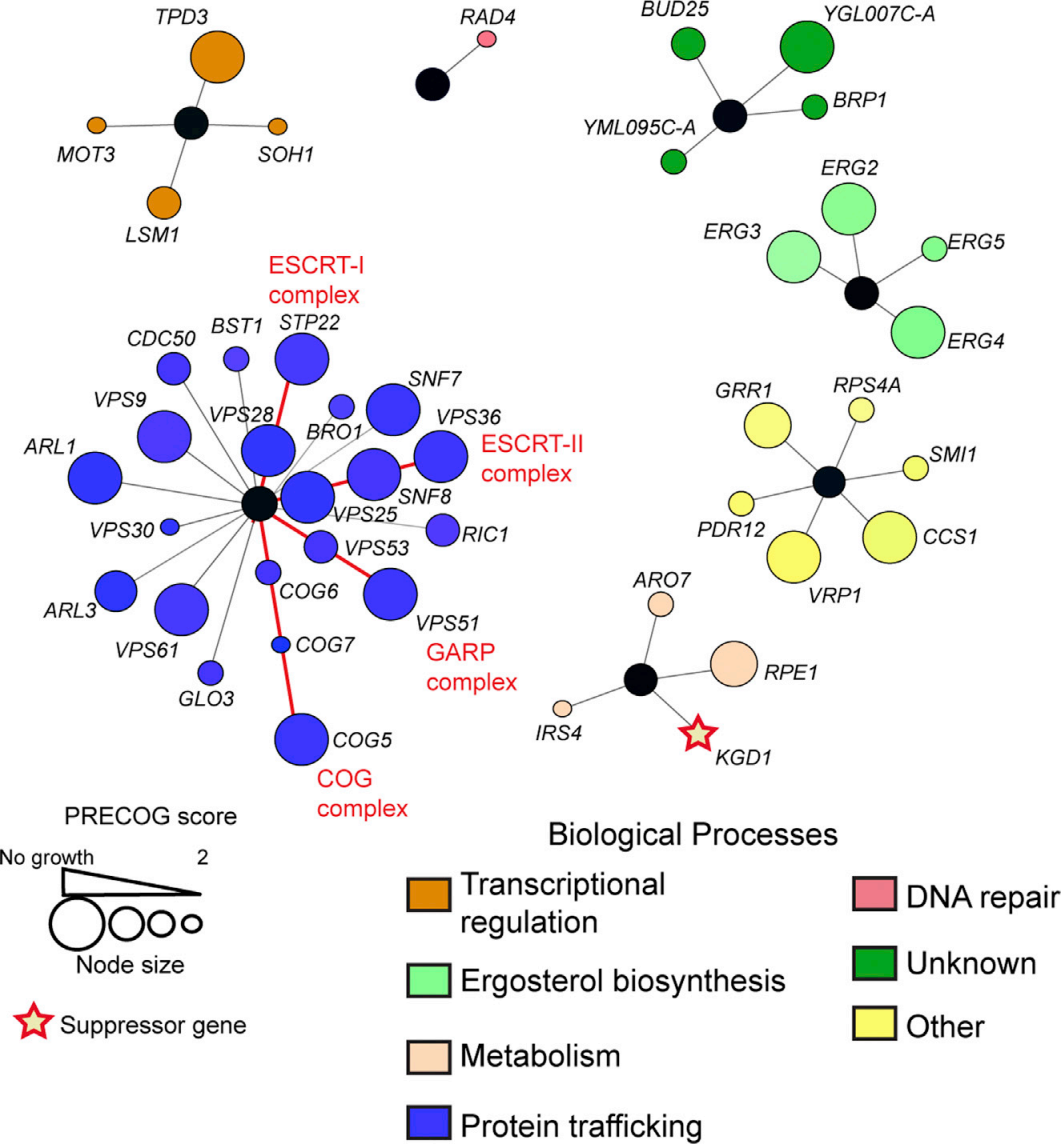
Network plot shows the adipic acid chemogenomic profile of *S*. *cerevisiae* The colored nodes depict genes that result in adipic acid sensitivity in yeast when deleted. The different genes are clustered based on color-coded biological processes. Adipic acid sensitivity resulting from gene deletion based on calculated PRECOG scores is shown by the node sizes. The suppressor gene is indicated by a star. Gene nodes connected by a red line represent genes encoding components of a protein complex as indicated. See also Figure S1.

Gene Ontology (GO) enrichment analysis (Robinson et al., 2002) identified numerous biological processes that were enriched in the screen including the ergosterol biosynthesis pathway (p = 1.147 × 10^−5^ (Table S1; Figure 1). Indeed, strains with an *ERG2*, *ERG3,* or *ERG4* deletion were hypersensitive to adipic acid and showed no growth in the presence of 120 mM adipic acid, suggesting that ergosterol biosynthesis is required for growth in adipic acid (Figure S1). The most represented biological process that modulates adipic acid toxicity in our screen is protein transport and trafficking (p = 3.80 × 10^−11^; Table S1 and Figure 1). In particular, genes encoding proteins within the ESCRT pathway such as *STP22-VPS29* (ESCRT-I), *VPS25-SNF8-VPS36* (ESCRT-II), and *SNF7* (ESCRT-III) were essential for growth in the presence of 120 mM adipic acid. Indeed, all these ESCRT pathway mutants failed to grow in the presence of 120 mM adipic acid. Deletion of Bro1, an auxiliary protein in the ESCRT pathway, which binds to *SNF7* (Wemmer et al., 2011), also results in sensitivity to adipic acid. Furthermore, deleting genes encoding protein complexes such as *VPS51-VPS53* (GARP complex) and *COG5-COG6-COG7* (COG complex) results in significant growth inhibition in the presence of adipic acid (Figure 1). Taken together, ergosterol biosynthesis and protein trafficking are essential biological processes required for yeast growth in adipic acid as deletion of key genes in these processes results in hypersensitivity to adipic acid.

### *KGD1* in the tricarboxylic acid cycle suppresses adipic acid toxicity

**Figure 2:**
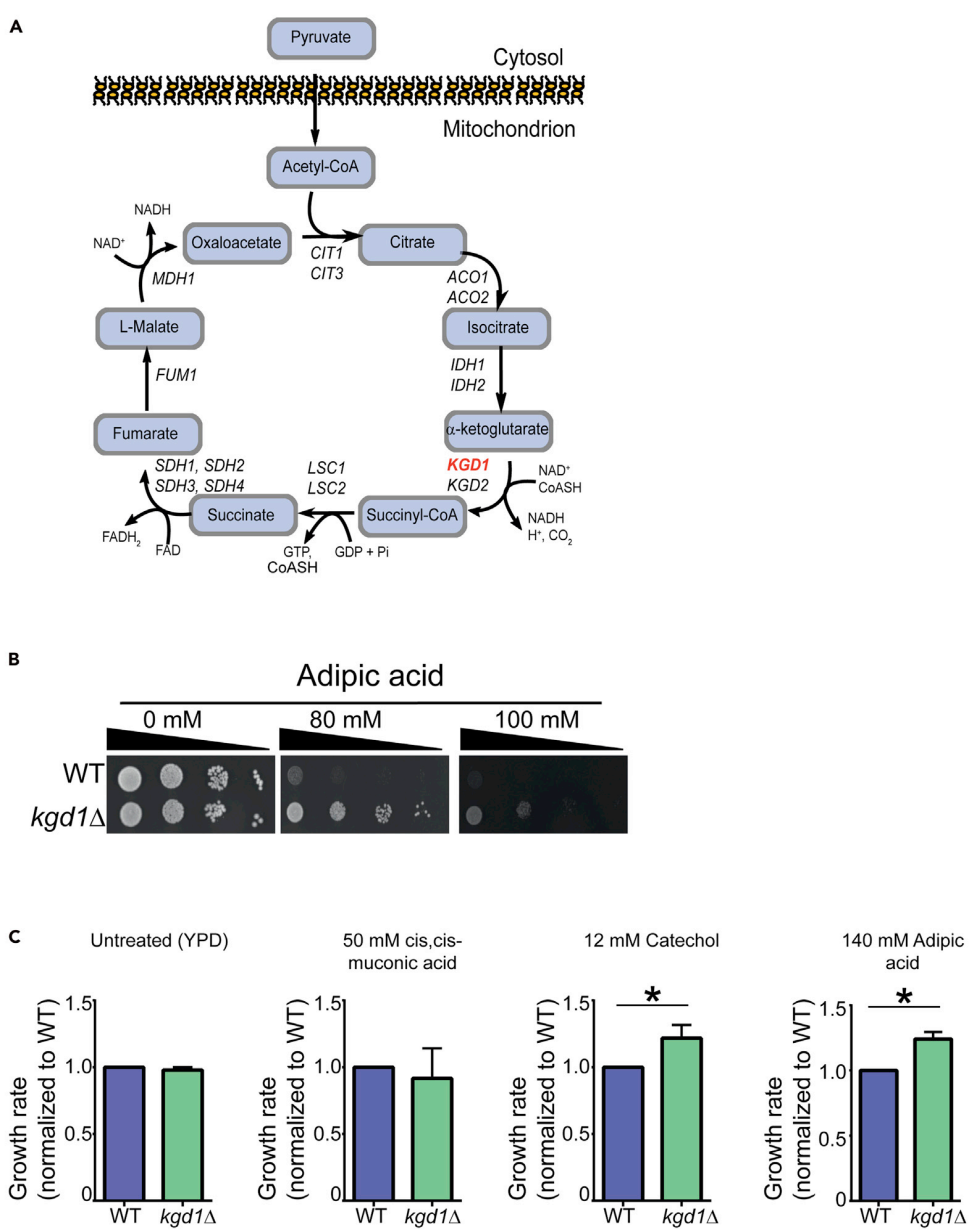
*KGD1* deletion suppresses adipic acid toxicity in yeast (A) Schematic of the tricarboxylic acid (TCA) cycle. (B) Dot assays were performed by spotting 4-fold serial dilutions of mid-log phase wild-type and *kgd1Δ* (YKB4943) cultures onto YPD agar plates supplemented with increasing concentrations of adipic acid and incubated at 30°C for 48 h. The images are representative of three biological replicates. (C) Wild-type (WT) and *kgd1Δ* (YKB4943) strains were grown to the mid-log phase; diluted to an OD_600_ of 0.1 in liquid YPD cultures with or without 12 mM catechol, 50 mM muconic acid, or 140 mM adipic acid; and automated growth curves were obtained in triplicates using the Bioscreen at 30°C. Growth rates of *kgd1Δ* were calculated for each treatment and normalized to that of the WT. Error bars represent 1 standard deviation. ∗p < 0.05. See also Figure S2.

The only adipic acid suppressor mutant identified was *KGD1*, which encodes an enzyme (α-ketoglutarate dehydrogenase) that converts α-ketoglutarate to oxaloacetate (Repetto and Tzagoloff, 1989) in the tricarboxylic acid (TCA) cycle (Figure 2A). We confirmed that the *kgd1Δ* strain grew better than the wild type during exposure to adipic acid in both liquid and solid YPD media (Figures 2B and S1A). Through liquid growth assays, we next investigated the involvement of other genes in the TCA cycle in modulating adipic acid tolerance. Apart from *sdh1Δ,* which is hypersensitive to adipic acid, all the other TCA cycle mutants grew similarly as the wild-type strain in adipic acid (Figure S2B).

Next, we asked if deleting *KGD1* also improved tolerance to catechol and *cis*,*cis*-muconic acid, two precursors in the adipic acid biosynthetic pathway. Therefore, we calculated yeast growth rates in liquid cultures containing 12 mM catechol or 50 mM *cis*,*cis*-muconic acid and compared it with that grown in YPD without any chemicals added. Although the *kgd1Δ* strain grew better than the wild type in media containing catechol (p < 0.05), the mutant strain grew similarly as the wild-type strain in media containing *cis*,*cis*-muconic acid (Figure 2C), suggesting that *KGD1* deletion confers cross-resistance to some precursors in the adipic acid biosynthesis pathway. In summary, *KGD1,* and not the TCA cycle, modulates adipic acid tolerance in yeast. Furthermore, deletion of *KGD1* also improved tolerance to catechol, a toxic adipic acid precursor.

### Genes encoding enzymes in the last four steps of the ergosterol biosynthesis pathway are required for growth in adipic acid

**Figure 3:**
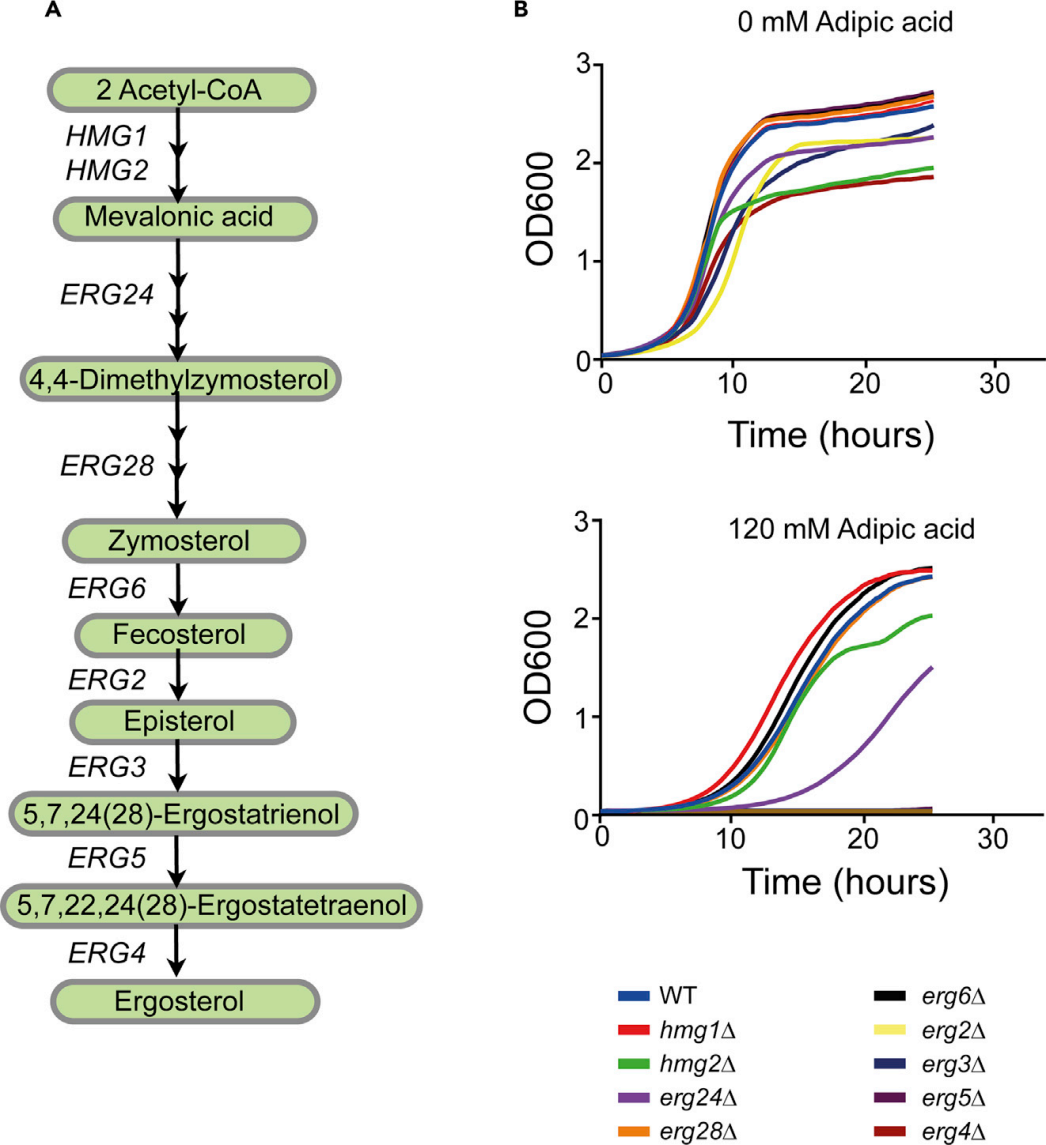
Last steps in the ergosterol biosynthesis pathway are essential for yeast growth in adipic acid (A) Schematic of the ergosterol biosynthesis pathway showing the nine non-essential genes in the pathway. (B) Wild-type (WT), *hmg1Δ* (YKB4956), *hmg2Δ* (YKB4957), *erg24Δ* (YKB4958), *erg6Δ* (YKB4959), *erg28Δ* (YKB4960), *erg2Δ* (YKB4874), *erg3Δ* (YKB4875), *erg5Δ* (YKB4877), and *erg4Δ* (YKB4876) cultures were grown to the mid-log phase, diluted to an OD_600_ of 0.1 in liquid YPD cultures with or without 120 mM adipic acid, and automated growth curves were obtained in triplicates using the Bioscreen at 30°C. The growth curves shown represent an average of three replicates. See also Figure S1.

To determine whether the ergosterol biosynthesis pathway (Figure 3A) modulates adipic acid toxicity, using liquid growth analysis we tested the sensitivity of all nine non-essential ergosterol pathway deletion mutants to adipic acid (*HMG1*, *HMG2*, *ERG24*, *ERG6*, *ERG28*, *ERG2*, *ERG3*, *ERG5*, *ERG4*) (Bhattacharya et al., 2018). These included the last four genes in the ergosterol biosynthesis pathway that were identified in our chemogenomic screen (*ERG2*, *ERG3*, *ERG5,* and *ERG4*, Figure 1). Similar to our chemogenomic screen, whereas *erg5Δ* cells display a mild sensitivity to adipic acid, *erg2Δ*, e*rg3Δ,* and *erg4Δ* cells showed a complete growth inhibition in media containing 120 mM adipic acid (Figure 3B). However, whereas *hmg2Δ* cells have minor sensitivity to adipic acid at higher OD, the other non-essential ergosterol pathway deletion mutants cells' growth profile was similar to those of wild-type cells upon 120 mM adipic acid treatment (Figure 3B).

**Figure 4:**
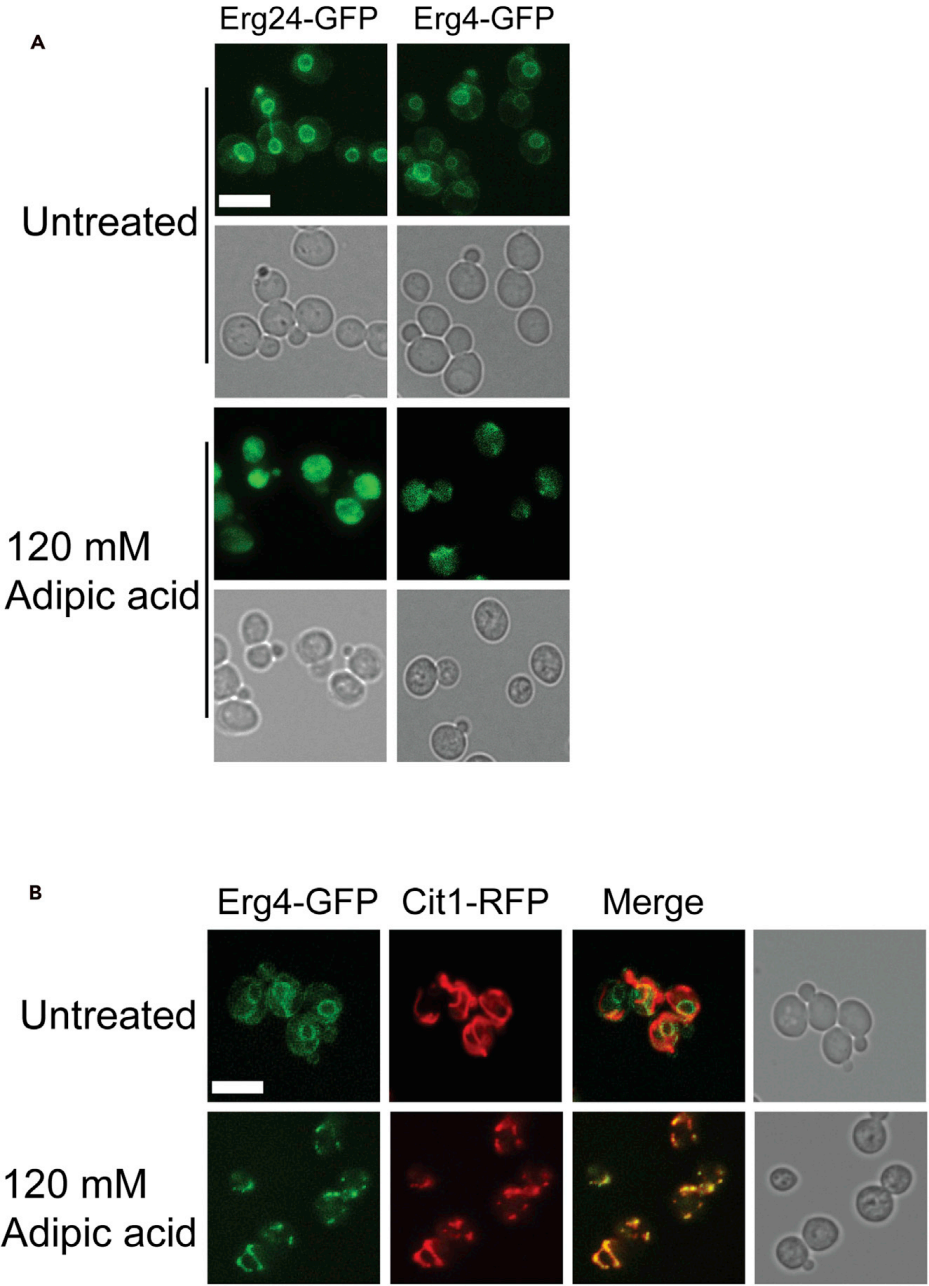
Ergosterol biosynthetic enzymes Hmg1, Erg4, and Erg24 change localization upon adipic acid treatment (A) Cells containing GFP fusions to Hmg1 (YKB4947), Erg4 (YKB5088), and Erg24 (YKB4951) were grown to mid-log phase, centrifuged, and resuspended in YPD media with or without adipic acid and grown at 30°C for 2 h and imaged. (B) Adipic acid treatment causes mitochondrial fragmentation and Erg4-GFP relocalization to the mitochondrial marker Cit1-RFP. Cells endogenously expressing Erg4-GFP and Cit1-RFP (YKB5091) were grown to mid-log phase, centrifuged, and resuspended in YPD media with or without adipic acid and grown at 30°C for 2 h before being imaged. Scale bar, 10 μm. Images are representative of three biological replicates. See also Figures S3–S5.

In order to delve deeper into the role of the ergosterol pathway in modulating adipic acid toxicity, including the essential pathway proteins, we next asked if 2 h of adipic acid treatment impacted the protein levels and/or subcellular localization of ergosterol pathway enzymes tagged with a green fluorescent protein (GFP). We did not observe any significant changes in protein level of any of the GFP-tagged ergosterol pathway enzymes upon adipic acid treatment (Figures S3 and S4). This suggests that even though the ergosterol biosynthesis pathway, particularly the last four steps, is required for growth in adipic acid, the pathway is not induced in the presence of the compound. However, two of the enzymes in the pathway changed localization upon adipic acid treatment. The endoplasmic reticulum (ER) localized Erg24-GFP (Jordá and Puig, 2020) and Erg4-GFP (Srinivas et al., 2019) moved to the cytosol upon adipic acid treatment (Figure 4A). Erg4-GFP also changed localization to punctate structures (Figure 4A). As Erg4 is the last enzyme in the pathway leading to the formation of ergosterol and its deletion results in hypersensitivity to adipic acid, we did a time course experiment to track changes in localization of Erg4-GFP. To this end, we grew cells expressing Erg4-GFP to log phase in YPD before adding 120 mM adipic acid, and localization was assessed at 0, 15, 30, and 60 min. We found that Erg4-GFP changed localization to punctate structures as early as 15 min of adipic acid exposure (Figure S5). As ergosterol is a major component of mitochondrial membranes (Krumpe et al., 2012) and decreases in pH can cause fragmentation of mitochondria (Pereira et al., 2010), using the mitochondrial marker Cit1-RFP, we asked if Erg4-GFP was relocalizing to mitochondria upon adipic acid treatment. As expected, in untreated cells, the ER-localized Erg4-GFP did not co-localize with Cit1-RFP (Figure 4B). Adipic acid treatment caused fragmentation of the mitochondria, and Erg4-GFP co-localized with Cit1-RFP. Together, these data suggest that the last four steps in the ergosterol biosynthesis pathway that convert fecosterol to ergosterol, although not induced during adipic acid treatment, are required for yeast growth during exposure to adipic acid. Indeed, key enzymes such as Hmg1, which catalyzes a rate-limiting step of the pathway (Bhattacharya et al., 2018), and Erg4, which catalyzes the final step in the pathway, change localization upon adipic acid treatment, suggesting a potential regulation of the pathway during growth in adipic acid.

### Ergosterol and Pdr12 efflux play non-overlapping roles protecting the cell from adipic acid toxicity

**Figure 5:**
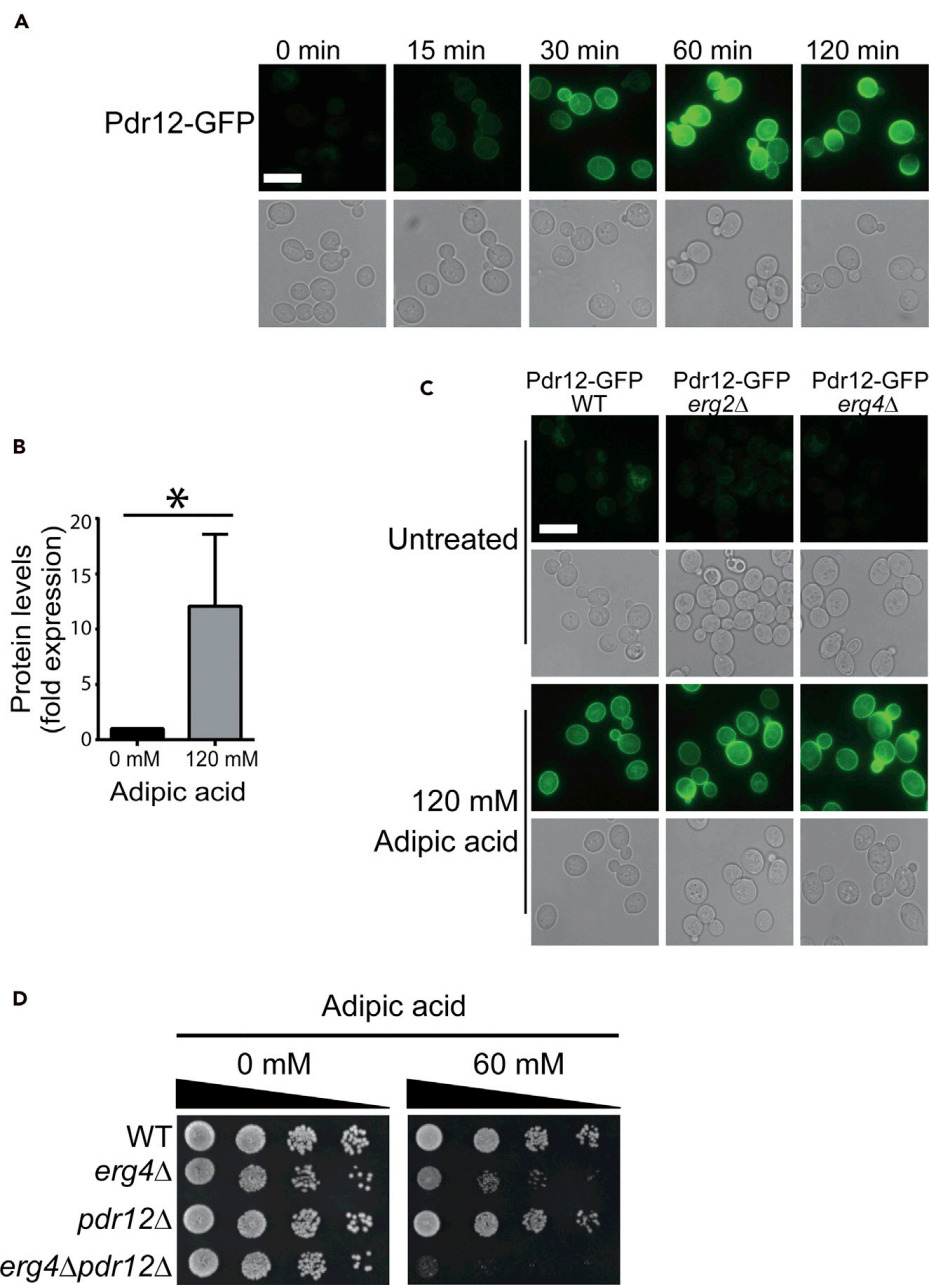
Adipic acid induction and plasma membrane localization of Pdr12 is not dependent on ergosterol (A and B) Pdr12-GFP is induced upon adipic acid treatment. (A) Wild-type cells expressing endogenously tagged Pdr12-GFP (YKB4889) were grown to mid-log phase, centrifuged and resuspended in YPD media with or without adipic acid, grown at 30°C, and images taken at the indicated time points. (B) Protein levels of Pdr12-GFP in YPD with and without adipic acid treatment for 30 min were quantitated using western blots. ∗Statistical significance at p < 0.05. Error bars represent 1 SD. (C) Deletion of *ERG2* and *ERG4* does not impact the adipic acid induction and plasma membrane localization of Pdr12-GFP. Wild-type (WT), *erg2Δ,* and *erg4Δ* strains expressing endogenously tagged Pdr12-GFP were grown to mid-log phase, centrifuged and resuspended in YPD media with or without adipic acid, and grown at 30°C for 1 h before imaging. Scale bar, 10 μm. (D) Synergistic effects of *erg4Δ* and *pdr12Δ* on adipic acid toxicity. Wild-type (WT), *erg4Δ* (YKB4876), *pdr12Δ* (YKB4383), and *erg4Δpdr12Δ* (YKB5036) were grown to mid-log phase in YPD medium before being diluted into fresh medium to an OD_600_ of 0.1. Four 10-fold serial dilutions were spotted onto YPD agar plates with or without adipic acid (60 mM) and incubated at 30°C for 48 h. The image is representative of three biological replicates. See also Figure S6.

Plasma membrane ergosterol has been implicated in the localization and activity of many plasma membrane transporters (Bagnat et al., 2000; Kodedová and Sychrová, 2015). Hence, we next sought to determine if the hypersensitivity of ergosterol biosynthetic pathway mutants to adipic acid reflected defects in Pdr12, the only plasma membrane transporter identified in our chemogenomic screen (Figures 1 and S6). As the overexpression of *PDR12* improves adipic acid tolerance (Pereira et al., 2019), we first asked if adipic acid treatment changed the protein levels or localization of Pdr12. We C terminally tagged endogenous *PDR12* with GFP (Pdr12-GFP) and assessed the impact of adipic acid treatment on protein levels and localization. Upon 120 mM adipic acid exposure, there was a rapid induction of Pdr12-GFP, which localized to the plasma membrane (Figure 5A). The induction of the protein was observed as early as 15 min after exposure to adipic acid (Figure 5A). Using quantitative western blots we found that Pdr12-GFP protein levels were induced 12-fold after 30 min of adipic acid treatment (Figure 5B). These data indicate that Pdr12 expression is increased during growth in adipic acid potentially to facilitate export of cytosolic adipic acid anions (adipate) in order to reduce intracellular toxicity.

As ergosterol has been reported to play a role as lipid raft in transporting newly synthesized proteins from the Golgi to the plasma membrane (Bastos et al., 2012; Mollinedo, 2012), we asked if ergosterol biosynthesis genes *ERG2* and *ERG4* were required for either the induction or localization of Pdr12-GFP to the plasma membrane upon adipic acid treatment. Wild-type, *erg2Δ,* and *erg4Δ* cells expressing endogenously tagged Pdr12-GFP were grown to midlog phase before being treated with 120 mM adipic acid for 1 h. We found that Pdr12-GFP in the ergosterol mutants was induced and localized to the plasma membrane similar to wild type, suggesting that Pdr12 localization to the plasma membrane is independent of ergosterol lipid rafts (Figure 5C). Although ergosterol may not be needed to localize Pdr12 to the plasma membrane, it may still be required for Pdr12 activity. If this was the case we would hypothesize that an *erg4Δpdr12Δ* sensitivity to adipic acid would be epistatic or similar to the single mutants. If, however, these two pathways were working in parallel, the double mutant would have increased sensitivity to adipic acid compared with the single mutants. Wild-type, *erg4Δ*, *pdr12Δ,* and *erg4Δpdr12Δ* cells were tested for growth on YPD agar plates with and without a sublethal dosage of adipic acid (Figure 5D). Although 60 mM adipic acid treatment causes slow growth of *erg4Δ*, it has limited impact on *pdr12Δ* cells. In contrast, the ability of *erg4Δpdr12Δ* cells to grow on adipic acid is dramatically slower than either *erg4Δ* or *pdr12Δ* cells. The synergistic sensitivity to adipic acid suggests that ergosterol and Pdr12 play independent roles in protecting the cells from adipic acid toxicity (Figure 5D). Taken together, these data suggest that although Pdr12 is rapidly induced upon adipic acid treatment, its localization to the plasma membrane is not mediated by ergosterol lipid rafts. Furthermore, our genetics suggests that Erg4, and presumably the ergosterol biosynthesis pathway, and Pdr12 play non-overlapping roles in buffering adipic acid toxicity.

### Adipic acid disrupts the Membrane Compartment of Can1 (MCC) and induces endocytosis

**Figure 6:**
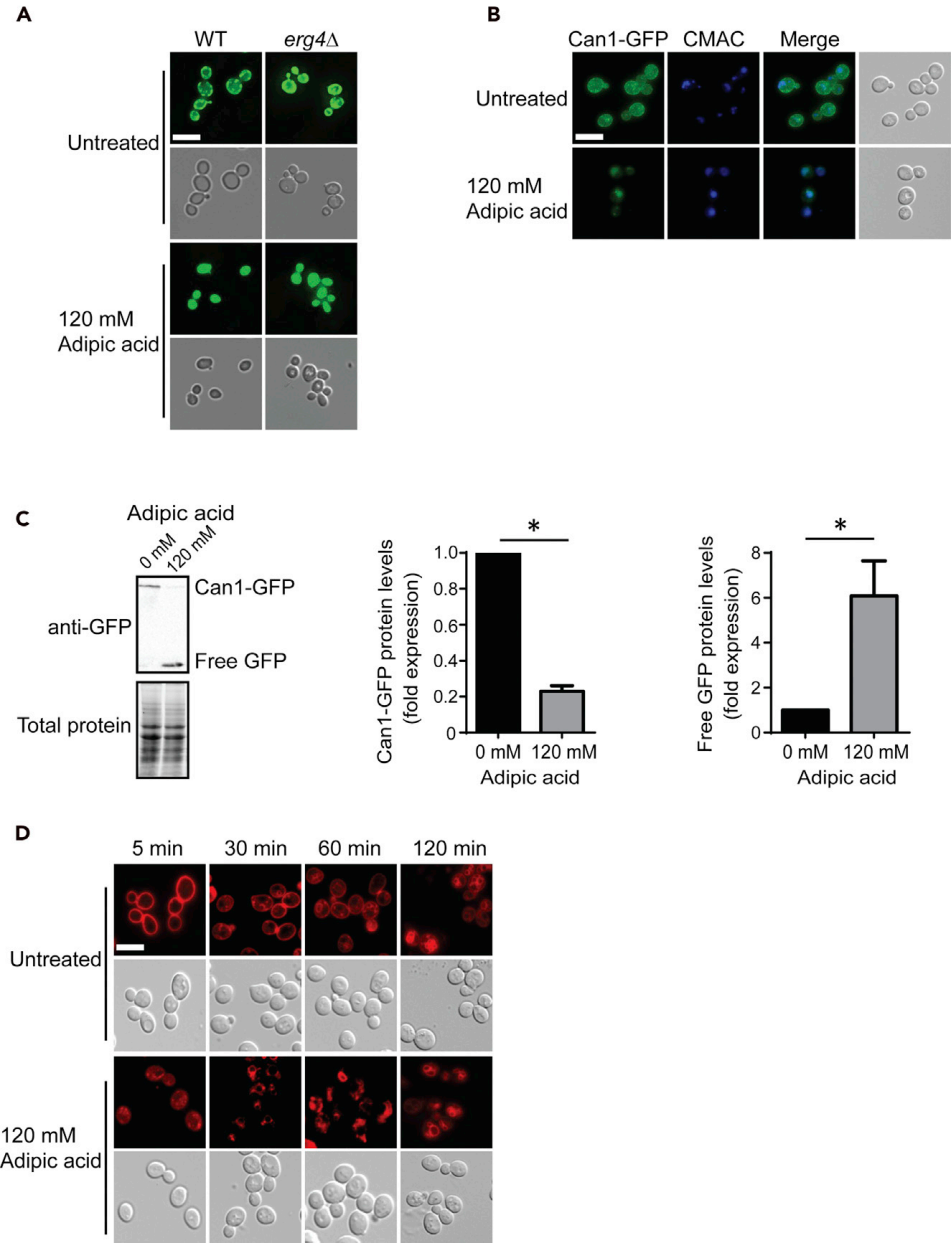
Adipic acid disrupts the MCC and induces endocytosis (A) Adipic acid treatment disrupts ergosterol plasma membrane patches. Mid-log phase cultures of wild-type (WT, YKB1079) and *erg4Δ* (YKB4876) cells were stained with the filipin dye and treated with or without 120 mM adipic acid for 1 h, before imaging. Scale bar, 10 μm; images are representative of three biological replicates. (B) Adipic acid treatment results in Can1-GFP relocalization to the vacuole. Wild-type cells endogenously expressing Can1-GFP (YKB5097) were grown to the mid-log phase in YPD media. Cells were then stained with the vacuole lumen dye CMAC for 15 min before being treated with 120 mM adipic acid for 1 h at 30°C after which they were imaged. Scale bar, 10 μm; images are representative of three biological replicates. (C) Can1-GFP is degraded upon adipic acid treatment. Quantitative western blot analysis were performed on whole-cell protein extracts, which had been collected by TCA extraction from Can1-GFP yeast cultures that were untreated (0 mM) or treated with adipic acid (120 mM) for 1 h. The anti-GFP blot is shown with the corresponding total protein for each lane as assessed using the Bio-Rad TGX system (left panel). The abundance of full-length Can1-GFP protein and free GFP was quantified relative to total protein and normalized to untreated levels (fold expression) (right panels). n = 3, ∗p < 0.05, error bars represent 1 SD. (D) Adipic acid treatment induces endocytosis. Mid-log phase cultures of the wild-type yeast (YKB1079) were stained with the FM4-64 and treated with or without adipic acid. Samples from the cultures were taken at 5, 30, 60, and 120 min after adipic acid treatment. The cells were centrifuged, resuspended in SC medium, and imaged. Scale bar, 10 μm; images are representative of three biological replicates. See also Figure S7.

To further probe the potential roles of ergosterol in adipic acid protection, we asked if the ergosterol-rich MCC was affected by adipic acid. MCCs are membrane microdomains and occur as one of several large membrane compartments on the plasma membrane (Malinsky et al., 2010). They also serve as membrane reservoirs of specific proteins such as the proton symports Can1, Tat2, and Fur4 as well as other proteins with unknown functions including Sur7, Fmp45, and Yn1194c (Douglas et al., 2011). Can1 is an arginine transporter that interacts with ergosterol in the MCC and also serves as an MCC marker (Grossmann et al., 2008). Not only is ergosterol required to target some of these membrane proteins including Can1 and Tat2 to the MCC but also ergosterol accumulates in the MCC, which is visualized as patches when cells are stained with the filipin dye (Grossmann et al., 2007; Malínská et al., 2003). Indeed, deletion of ergosterol biosynthesis genes has been shown to impair MCC formation (Grossmann et al., 2006). To assess the effect of adipic acid on the MCC, wild-type and *erg4Δ* cells were stained with filipin before treatment with adipic acid. As expected in untreated wild-type cells, filipin staining of the plasma membrane appeared as punctate structures or MCC patches, whereas in *erg4Δ* cells punctate filipin staining is disrupted (Figure 6A). Upon adipic acid treatment, the filipin-stained MCC patches on the plasma membrane were lost in wild-type cells (Figure 6A), suggesting that adipic acid impacts the plasma membrane MCC. As a secondary method to assess the impact of adipic acid on the MCC, we utilized the MCC marker Can1-GFP, and as expected during growth in adipic acid, Can1-GFP is lost from the plasma membrane and is enriched in punctate internal structures (Figures 6B and S7). Previous studies have reported that Can1-GFP undergoes endocytosis to the vacuole when it is lost from the plasma membrane (Ghaddar et al., 2014; Gournas et al., 2017). Using two vacuolar markers, CMAC and FM4-64, which stain the lumen of the vacuole and the vacuolar membrane, respectively, we determined that Can1-GFP localized to the vacuoles upon adipic acid treatment (Figures 6B and S7). Furthermore, upon adipic acid treatment there is a significant decrease in full-length Can1-GFP protein levels and an increase in free GFP (Figure 6C). Together this suggests that adipic acid exposure disperses Can1 from the MCC, which is subsequently internalized by endocytosis to the vacuole for degradation.

As the MCCs are sites for endocytosis on the plasma membrane, we hypothesized that adipic acid treatment induced endocytosis as a mechanism to potentially downregulate membrane transporters that import adipic acid into the cell, which results in accumulation and toxicity. To test this hypothesis, we utilized the vacuole dye FM4-64, which is also commonly used to measure endocytosis (Brach et al., 2014). We performed a time course experiment and determined that adipic acid-treated cells internalized FM4-64 faster than untreated cells (Figure 6D). Remarkably, instead of diffused plasma membrane staining at time point 0 min, FM4-64 is already being internalized in adipic acid-treated cells, and vacuolar staining of cells by FM4-64 was visible after 15 min of incubation with adipic acid, whereas it took up to 120 min to observe clear vacuolar staining in the untreated cells. Together, these data indicate that adipic acid disrupts the membrane component of Can1 potentially by an increased rate of endocytosis.

## Discussion

Overcoming the challenge of adipic acid toxicity in yeast is imperative to achieve high titers of the acid in production through yeast fermentation. Therefore, in this study we sought to identify the mechanisms underlying the toxic effect of the adipic acid in yeast and identify genes that could be targeted to improve tolerance in yeast. Toward this goal, we performed a chemogenomic screen and systematically assessed the impact of each non-essential gene on adipic acid tolerance. Our screen revealed that a disruption in protein trafficking and the ergosterol biosynthesis pathway resulted in significant increase in sensitivity to adipic acid. On the contrary, deleting *KGD1*, a gene in the TCA cycle, resulted in improved tolerance to adipic acid.

### How is KGD1 associated with enhanced tolerance to adipic acid?

In our screen, we identified one suppressor gene whose deletion resulted in tolerance to adipic acid. To the best of our knowledge, this is the first study reporting the role of the TCA cycle gene, *KGD1*, in increasing tolerance to adipic acid and its precursor catechol (Figure 2). Surprisingly, only the deletion of *KGD1,* but not other genes in the TCA cycle, improved adipic acid tolerance (Figures 2 and S2). How is this occurring? One possibility is that deleting *KGD1* results in the redirected metabolic flux toward acetyl-CoA production and this resulted in high amounts of acetate in the cell (Gruchattka and Kayser, 2015). Although we did not quantify intracellular levels of acetate in our *kgd1Δ* strain, future work should investigate if priming yeast cells with acetic acid before adipic acid exposure results in increased tolerance. This investigation will be worthwhile because acetic acid has also been shown in earlier reports to activate the plasma membrane transporter Pdr12 (Kim et al., 2019; Mira et al., 2010a). Given that Pdr12 overexpression improved tolerance to adipic acid (Pereira et al., 2019), intracellular levels of acetate in *kgd1Δ* strains may also result in Pdr12 induction and increased tolerance to adipic acid. If this was the case, however, one would anticipate that other mutants in the TCA cycle that increase cellular acetate may also confer adipic acid tolerance. However, these were not identified in the screen, potentially due to functional redundancy. Alternatively, Kgd1 may have biological functions outside of the TCA cycle. This is potentially the case because although *KGD1* is a component of the mitochondrial alpha-ketoglutarate dehydrogenase complex together with *KGD2* (Heublein et al., 2014), only *KGD1* is associated with adipic acid tolerance. It will be interesting to further investigate why deleting *KGD1* suppresses adipic acid toxicity.

### How does ergosterol modulate adipic acid toxicity?

Ergosterol and other yeast sterols have been reported to modulate different stresses in yeast (Caspeta et al., 2014; Fletcher et al., 2017; Liu et al., 2017). Interestingly, in the present study we establish that cellular ergosterol also plays a role in adipic acid tolerance. Here, we show that disrupting the final steps of the ergosterol biosynthetic pathway results in severe growth defects in yeast upon adipic acid treatment (Figures 3B and S1). It is likely that ergosterol mutants lacking genes in the final steps of the pathway have variations in their sterol composition. Previously, a study reported that yeast mutants lacking enzymes coded for by these ergosterol pathway genes accumulate a mixture of sterols because Erg2, Erg3, Erg4, and Erg5, which catalyze the last steps of the pathway, are promiscuous in their choice of substrate (Abe and Hiraki, 2009). Indeed, *erg2Δ* has been shown to accumulate fecosterol (Erg2 precursor) as well as ergosta-5,8,12-trienol and ergosta-8-enol, whereas *erg3Δ* accumulates a mixture of episterol, ergosta-7,22-dienol, and ergosta-7-enol (Abe and Hiraki, 2009; Johnston et al., 2020). On the other hand, *erg5Δ* accumulates ergosta-5,7,24(28)-trienol and ergosta-5,7-dienol, whereas *erg4Δ* mutant accumulates ergosta-5,7,22,24(28)-tetraen-3-ol (ergosterol precursor) (Abe and Hiraki, 2009). These sterol precursors will impact the physical properties of the cell membrane in a way different from the wild-type membrane, which is made up of ergosterol as the predominant sterol. Ergosterol is a flat sterol, whereas sterol precursors such as fecosterol and episterol are bent (Caspeta et al., 2014; Shahedi et al., 2006) and will alter the cell membrane by interfering with the van der Waals's interactions within the membrane's lipid bilayer (Grossmann et al., 2007). In the context of protecting cells from adipic acid toxicity, it is possible that the membrane structure maintained by ergosterol limits the rate at which the undissociated adipic acid diffuses across the plasma membrane into the cytosol from the extracellular environment.

As mentioned earlier, maintaining the right amount and composition of sterol is essential for growth in adipic acid. Changes in subcellular localization of sterol enzymes occur in cells as a way of regulating the activity of these enzymes (Leber et al., 1998). This might be the case for Erg24 and Erg4 during growth in adipic acid (Figure 4A) particularly because Erg4 catalyzes the final step of the pathway required for ergosterol biosynthesis. Alternatively, and not mutually exclusive, membrane sterol composition may be playing a critical role in the activity and/or localization of membrane-bound proteins. For example the activity of Pdr5, an ABC transporter reported to increase yeast tolerance to certain stresses, is impaired in ergosterol biosynthesis mutants (Kodedová and Sychrová, 2015). Similarly, ergosterol has been shown to be required for Pdr12 function, which is essential for tolerance to organic acids (Xu and Li, 2020). Yeast mutants that lack genes in the ergosterol biosynthesis pathway are sensitive not only to adipic acid but also to other organic acids such as sorbic acid (Mollapour et al., 2004). Although we did not investigate whether the efflux activity of Pdr12 is also significantly impaired in ergosterol mutants, we showed that deleting Pdr12 in an ergosterol mutant resulted in a synergistic effect on growth in adipic acid. The *erg4Δpdr12Δ* mutant was more sensitive to adipic acid than the *erg4Δ* or *pdr12Δ* alone (Figure 5D). This observation suggested that ergosterol plays other roles in modulating adipic acid toxicity beyond a potential role in regulating Pdr12. Regarding its role in the localization of membrane-bound proteins, ergosterol transports and targets membrane proteins such as Can1 and Tat2 to the MCC via ergosterol lipid rafts (Grossmann et al., 2008). Furthermore, ergosterol along with several proteins forms the MCC, which are sites of endocytosis on the plasma membrane, and defects in MCC formation results in impaired endocytosis (Heese-Peck et al., 2002). From our data, adipic acid impacts endocytosis by disrupting MCC formation, which was evidenced in the rapid internalization of Can1-GFP and FM4-64 (Figure 6D).

### Why is protein trafficking and vacuolar transport essential for yeast growth in adipic acid?

Nearly half of the genes in the adipic acid chemogenomic profile are associated with protein trafficking and vacuolar transport, suggesting that protein degradation and recycling in the vacuole is an essential biological process required for survival during yeast growth in adipic acid. Interestingly, most of these genes code for proteins that are components of the complexes that form the Endosomal Sorting Complexes Required for Transport (ESCRT) machinery. The ESCRT pathway is used by cells to degrade specific proteins on the plasma membrane by endocytosis (Wenzel et al., 2018). Intriguingly, our study shows that adipic acid impacts and disrupts the MCC (Figure 6A), which serves as a site for endocytosis and also provides a protective surrounding for several plasma membrane proteins and transporters including Can1 permease. A previous study has shown that when the protective area offered by the MCC is disrupted, proteins that reside in this compartment are released and become susceptible to cellular internalization and endocytosis (Grossmann et al., 2007, 2008). This explains why when adipic acid disrupts the MCC, Can1 rapidly changes localization to the vacuole for degradation (Figures 6B and 6C). This explanation was independently strengthened by the fact that adipic acid induced endocytosis (Figure 6D).

Endocytosis is essential for degrading and recycling of plasma membrane proteins. Specific membrane proteins are ubiquitinylated, internalized by endocytosis, and transported to the endosomes via the ESCRT pathway for vacuolar degradation (Feyder et al., 2015). Although organic acids are generally known to enter cells by passive diffusion, it has been shown that some plasma membrane transporters such as Jen1 and Acy1 are able to transport lactate, acetate, and other organic acids into the cell (Casal et al., 2008; McDermott et al., 2010). We speculate that endocytosis is induced in adipic acid-treated cells as a way of degrading non-specific membrane transporters that import extracellular adipic acid into the cell in order to prevent intracellular accumulation of the acid. It will be interesting to screen and identify non-specific transporters that import adipic acid into the cell and determine how adipic acid treatment impacts their degradation by endocytosis. Furthermore, as endocytosis is induced in adipic acid-treated cells as a survival mechanism, it is imperative that all the proteins involved in this process such as the ESCRT pathway proteins are maintained. Hence, when any of these key proteins are disrupted by deleting the genes that encode them, the cells become hypersensitive to adipic acid as shown in the adipic acid chemogenomic profile in this study.

### Conclusion

In conclusion, our study provides an outlook into the basic cellular mechanisms underlying adipic acid toxicity in yeast and provides potential genetic candidates that can be engineered for tolerance to boost adipic acid production in yeast. The results presented in this study highlight an induction of endocytosis in cells treated with adipic acid. It is therefore essential to maintain ergosterol biosynthesis because ergosterol is required to maintain the integrity of the plasma membrane as well as the MCC needed for endocytosis. Furthermore, *KGD1* deletion suppresses the toxicity of adipic acid and its toxic catechol precursor, making this gene an important target for engineering robust adipic acid production strains.

### Limitations of the study

The present study demonstrated an important role for the last four steps of the ergosterol biosynthesis pathway in protecting yeast cells from the toxic effects of adipic acid. Further studies will be required to determine if modulating the ergosterol pathway, such as overexpression, can improve growth upon adipic acid exposure. In addition, as this work was conducted in the laboratory yeast strain background S288c further studies are required to determine if the findings presented here are transferrable to industrial yeast strains.

### Resource availability

#### Lead contact

Further information and requests for resources and reagents should be directed to and will be fulfilled by the lead contact, Kristin Baetz (kbaetz@uottawa.ca).

#### Materials availability

This study did not generate new unique reagents. Yeast strains generated in this study that are not available commercially are available from the lead contact without restriction with the requestor paying for shipping costs.

#### Data and code availability

The published article includes all datasets generated or analyzed during this study.

## Methods

All methods can be found in the accompanying transparent methods supplemental file.

## Supplemental information

Document S1. Transparent methods, Figures S1–S7, and Table S2

Table S1. Summary of confirmed genetic hits in the chemogenomic screen and their associated biological processes, related to Figure 1
