# Supplementary material for: A yeast chemogenomic screen identifies pathways that modulate adipic acid toxicity
Source: iScience. 2021 Mar 18;24(4):102327. doi: 10.1016/j.isci.2021.102327 (PMC8050732; doi:10.1016/j.isci.2021.102327)
Supplement: Document S1. Transparent methods, Figures S1–S7, and Table S2 [file mmc1.pdf]

iScience, Volume *24*

Supplemental information

A yeast chemogenomic screen identifies pathways that modulate adipic acid toxicity

Eugene Fletcher, Kevin Mercurio, Elizabeth A. Walden, and Kristin Baetz

Supplemental Information

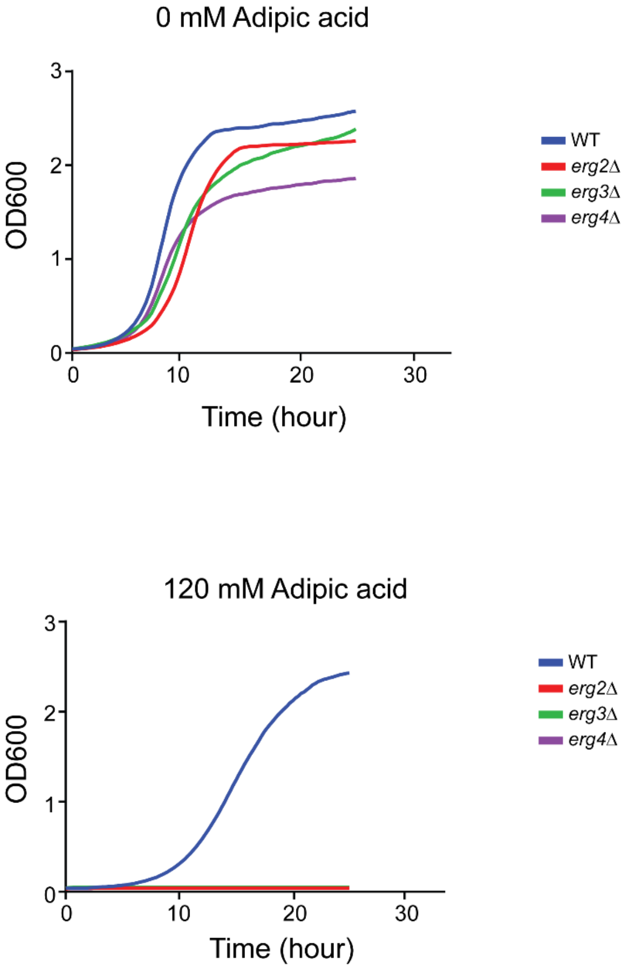

Figure S1: Deletion of genes in the late stage of the ergosterol biosynthesis pathway result in hypersensitivity to adipic acid, related to Figures 1 and 3. Wild type (WT), *erg2Δ*, *erg3Δ* and *erg4Δ* cultures were grown to the mid-log phase, diluted to an OD_{600} of 0.1 in liquid cultures with and without adipic acid and grown at 30 ^{o}C using a BioScreen. The automated growth curves were performed in triplicates.

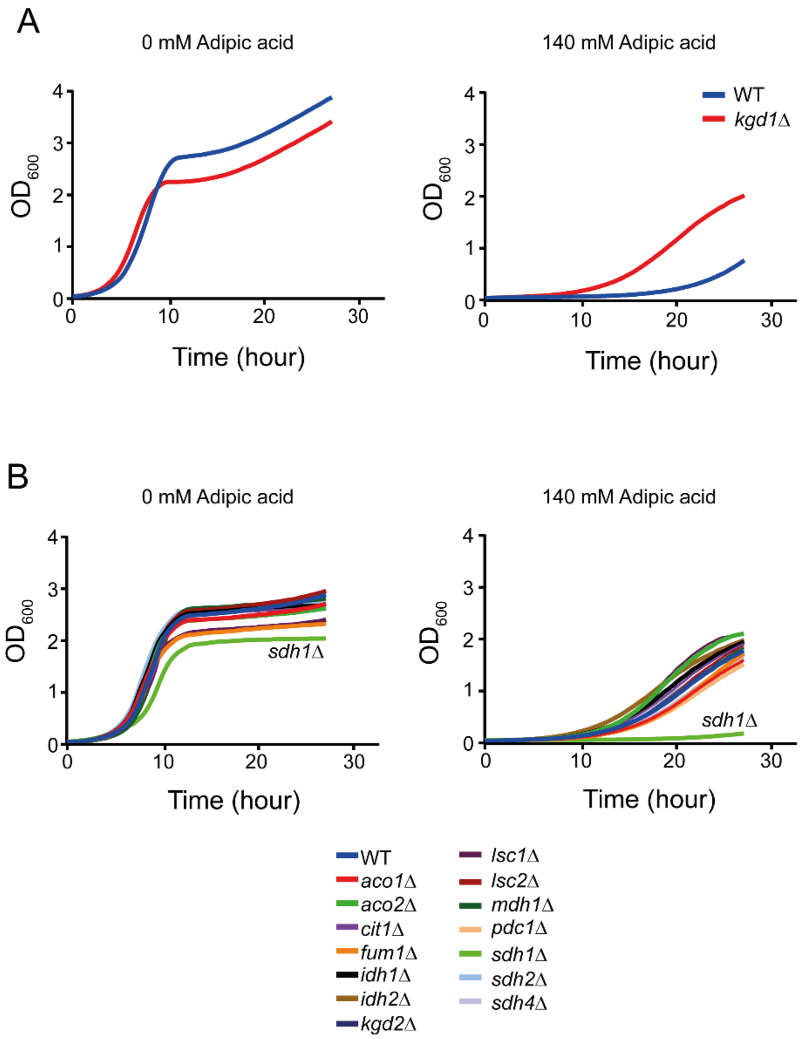

Figure S2: *KGD1* deletion suppresses adipic acid toxicity while other genes encoding TCA cycle enzymes do not modulate adipic acid toxicity, related to Figure 2. (A) WT and *kgd1Δ* cultures were grown to the mid-log phase, diluted to an OD_{600} of 0.1 in liquid cultures with or without adipic acid and grown at 30°C using a BioScreen. (B) WT, *aco1Δ* (YKB5099), *aco2Δ* (YKB5100), *cit1Δ* (YKB5101), *fum1Δ* (YKB5102), *idh1Δ* (YKB5103), *idh2Δ* (YKB5104), *kgd2Δ* (YKB5105), *lsc1Δ* (YKB5106), *lsc2Δ* (YKB5107), *mdh1Δ* (YKB5108), *pdc1Δ* (YKB5109), *sdh1Δ* (YKB5110), *sdh2Δ* (YKB5111) and *sdh4Δ* (YKB5112) cultures were grown to the mid-log phase, diluted to an OD_{600} of 0.1 in liquid cultures with or without adipic acid and grown at 30°C using a BioScreen. The automated growth curves were performed in triplicates.

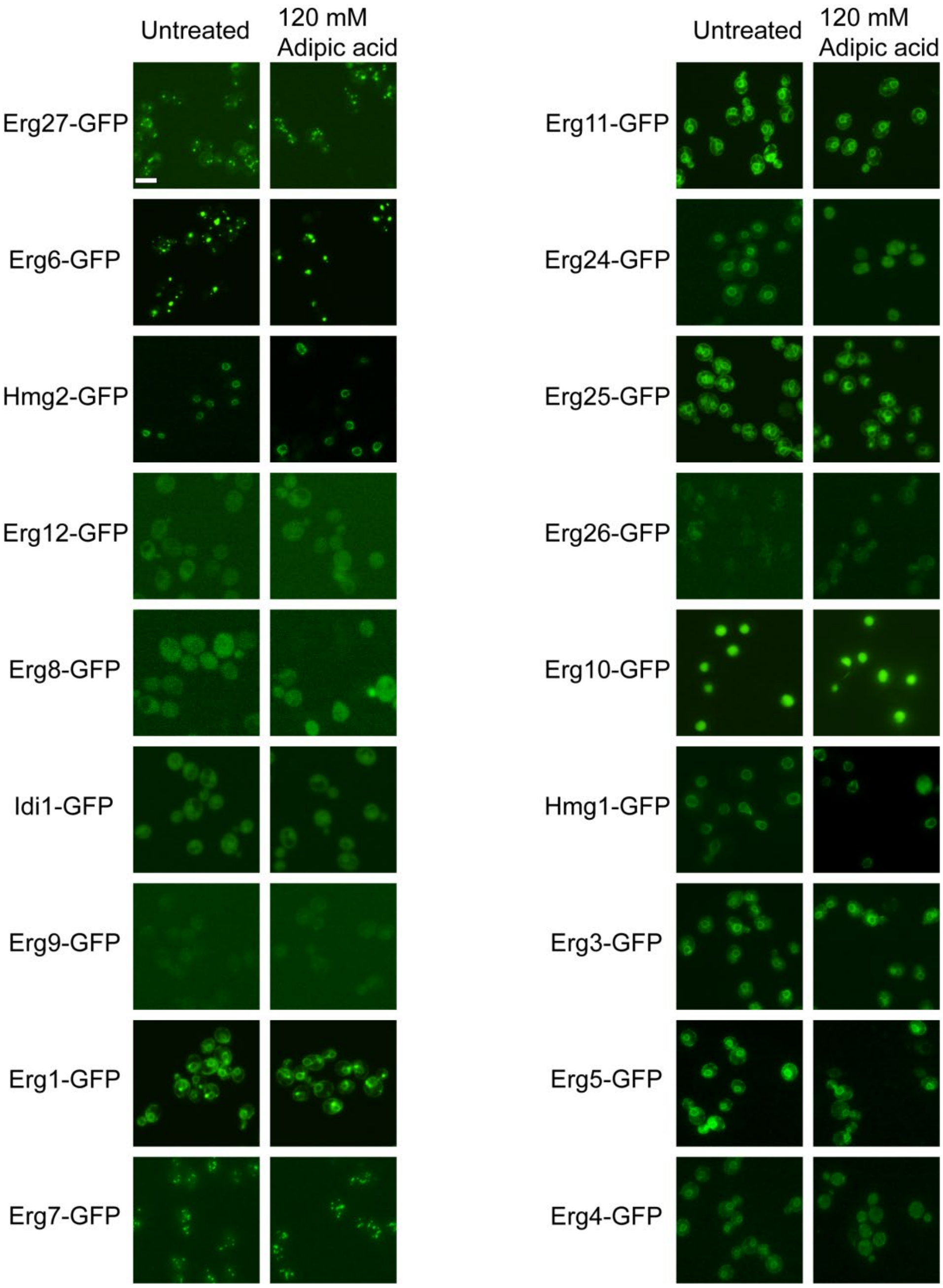

Figure S3: Ergosterol biosynthesis enzymes are not induced upon adipic acid treatment, related to Figure 4. Wild type (WT) cells expressing GFP-tagged ergosterol biosynthesis enzymes were grown to mid-log phase, centrifuged and resuspended in YPD media with or without adipic acid and grown at 30 ^{o}C. Green fluorescence of the cultures was measured after incubation for 2 hours.

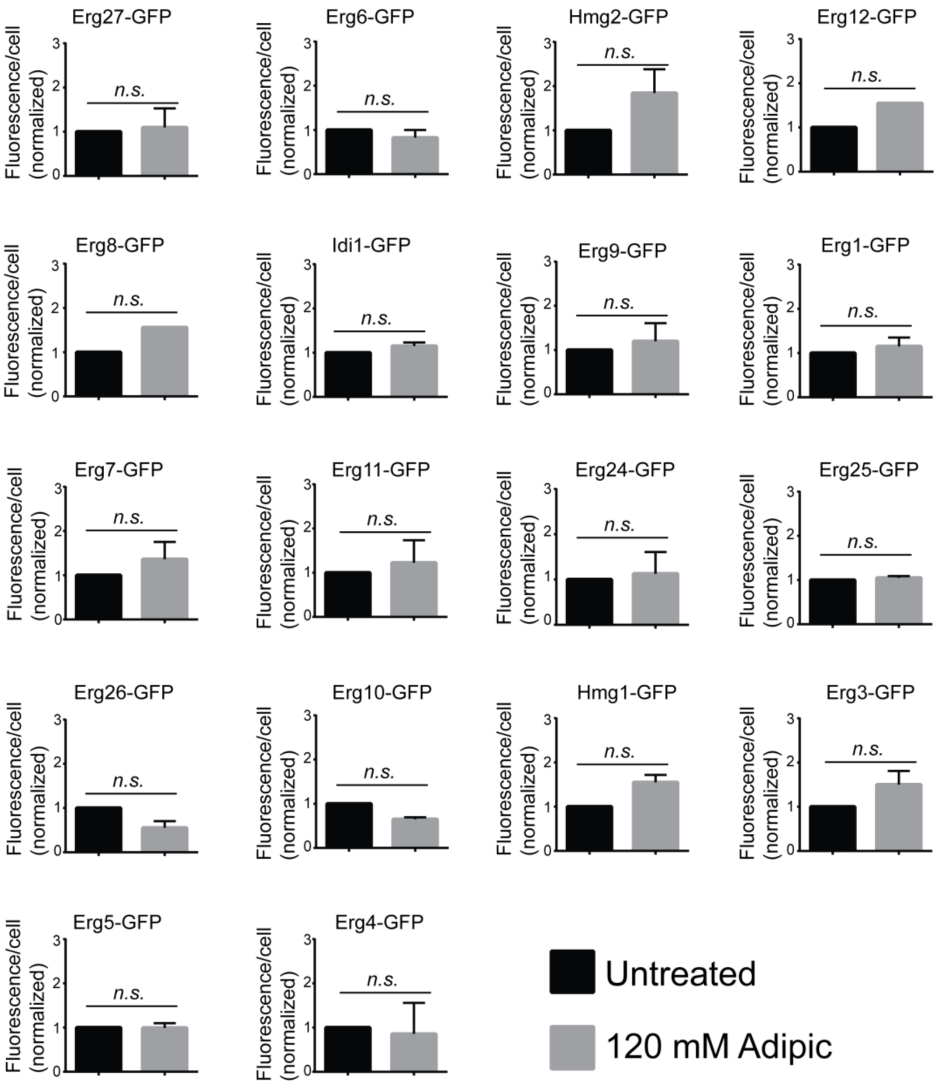

Figure S4: Ergosterol biosynthesis enzymes are not induced upon adipic acid treatment, related to Figure 4. Wild type (WT) cells expressing GFP-tagged ergosterol biosynthesis enzymes were grown to mid-log phase, centrifuged and resuspended in YPD media with or without adipic acid and grown at 30 ^{o}C. Green fluorescence of the cultures was measured after incubation for 2 hours. A minimum of 50 cells were captured for each image. Fluorescence intensity from cells were quantified using the ImageJ software and this was normalized to the untreated cultures for each strain tested. *n.s.* represents “not significant”. Error bars represent 1 STD.

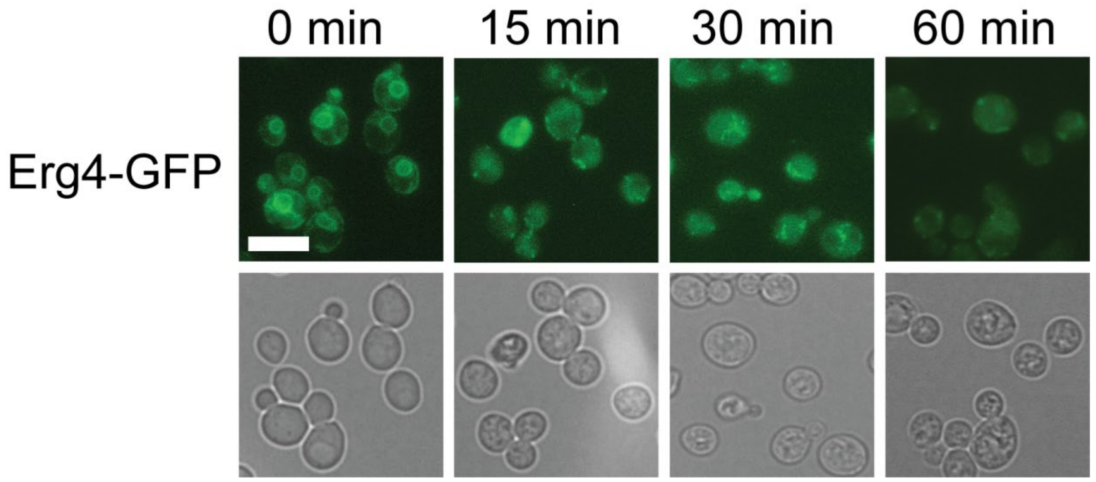

Figure S5: Ergosterol biosynthesis enzyme Erg4 changes localization over time upon adipic acid treatment, related to Figure 4. Cells expressing Erg4-GFP (YKB5088) were grown to mid-log phase, centrifuged and resuspended in YPD media with adipic acid and incubated at 30 ^{o}C. Images were taken at the start of the experiment (0 minutes) and after 15, 30 and 60 minutes of incubation with 120 mM adipic acid. The scale bar represents 10 µm. Images are representative of three biological replicates.

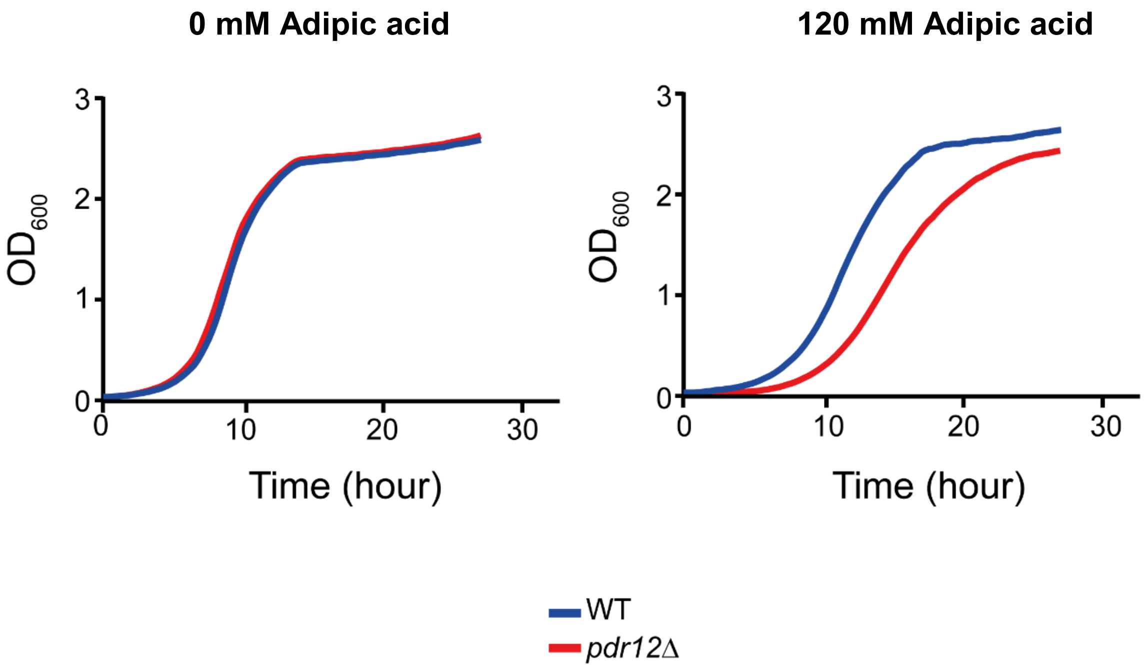

Figure S6: *PDR12* deletion increases yeast sensitivity to adipic acid, related to Figure 5. Wild type (WT) and *pdr12Δ* (YKB4383) cultures were grown to the mid-log phase, diluted to an OD_{600} of 0.1 in liquid cultures with and without adipic acid and grown at 30 ^{o}C using a BioScreen. The automated growth curves were performed in triplicates.

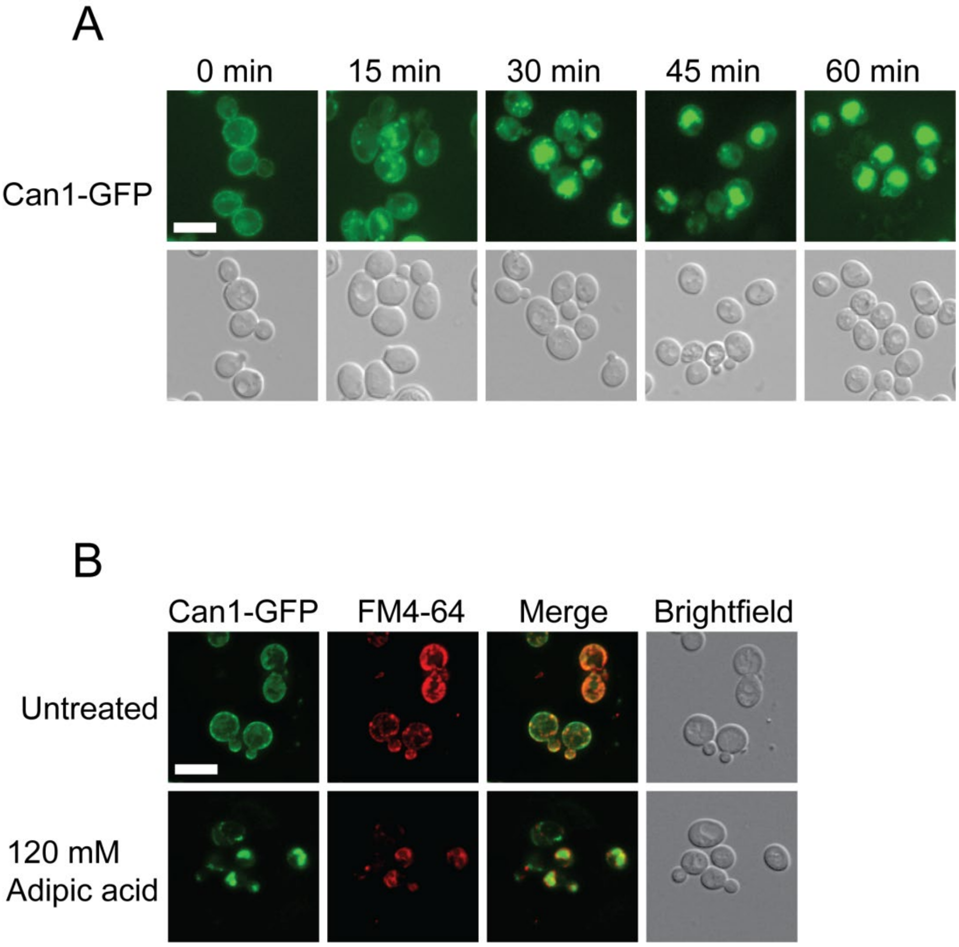

Figure S7: Can1-GFP changes localization from the plasma membrane to the vacuole upon adipic acid treatment, related to Figure 6. (A) Can1-GFP cells were grown to mid-log phase, centrifuged and resuspended in YPD media with or without adipic acid and grown at 30 ^{o}C.(B) The cells were then stained with the FM4-64 dye to visualize co-localization of Can1-GFP with the vacuole. FM4-64 (red) stains the vacuole membrane. Green and red fluorescence of the cultures were measured after incubation for 1 hour.

Table S2. Strains used in this study, related to Transparent Methods

Strains	Auxotrophies	Ref. or source
BY4741, YKB1079	*MATa his3Δ1 leu2Δ0 met15Δ0 ura3Δ0*	Open Biosystems
YKB4943	*MATa his3Δ1 leu2Δ0 met15Δ0 ura3Δ0* ***kgd1Δ***::*KANMX*	Open Biosystems
YKB5099	*MATa his3Δ1 leu2Δ0 met15Δ0 ura3Δ0* ***aco1Δ***::*KANMX*	Open Biosystems
YKB5100	*MATa his3Δ1 leu2Δ0 met15Δ0 ura3Δ0* ***aco2Δ***::*KANMX*	Open Biosystems
YKB5101	*MATa his3Δ1 leu2Δ0 met15Δ0 ura3Δ0* ***cit1Δ***::*KANMX*	Open Biosystems
YKB5102	*MATa his3Δ1 leu2Δ0 met15Δ0 ura3Δ0****fum1Δ***::*KANMX*	Open Biosystems
YKB5103	*MATa his3Δ1 leu2Δ0 met15Δ0 ura3Δ0* ***idh1Δ***::*KANMX*	Open Biosystems
YKB5104	*MATa his3Δ1 leu2Δ0 met15Δ0 ura3Δ0* ***idh2Δ***::*KANMX*	Open Biosystems
YKB5105	*MATa his3Δ1 leu2Δ0 met15Δ0 ura3Δ0* ***kgd2Δ***::*KANMX*	Open Biosystems
YKB5106	*MATa his3Δ1 leu2Δ0 met15Δ0 ura3Δ0* ***lsc1Δ***::*KANMX*	Open Biosystems
YKB5107	*MATa his3Δ1 leu2Δ0 met15Δ0 ura3Δ0* ***lsc2Δ***::*KANMX*	Open Biosystems
YKB5108	*MATa his3Δ1 leu2Δ0 met15Δ0 ura3Δ0****mdh1Δ***::*KANMX*	Open Biosystems
YKB5109	*MATa his3Δ1 leu2Δ0 met15Δ0 ura3Δ0* ***pdc1Δ***::*KANMX*	Open Biosystems
YKB5110	*MATa his3Δ1 leu2Δ0 met15Δ0 ura3Δ0* ***sdh1Δ***::*KANMX*	Open Biosystems
YKB5111	*MATa his3Δ1 leu2Δ0 met15Δ0 ura3Δ0* ***sdh2Δ***::*KANMX*	Open Biosystems
YKB5112	*MATa his3Δ1 leu2Δ0 met15Δ0 ura3Δ0* ***sdh4Δ***::*KANMX*	Open Biosystems
YKB4956	*MATa his3Δ1 leu2Δ0 met15Δ0 ura3Δ0****hmg1Δ***::*KANMX*	Open Biosystems
YKB4957	*MATa his3Δ1 leu2Δ0 met15Δ0 ura3Δ0****hmg2Δ***::*KANMX*	Open Biosystems
YKB4958	*MATa his3Δ1 leu2Δ0 met15Δ0 ura3Δ0****erg24Δ***::*KANMX*	Open Biosystems
YKB4959	*MATa his3Δ1 leu2Δ0 met15Δ0 ura3Δ0* ***erg6Δ***::*KANMX*	Open Biosystems
YKB4960	*MATa his3Δ1 leu2Δ0 met15Δ0 ura3Δ0****erg28Δ***::*KANMX*	Open Biosystems
YKB4874	*MATa his3Δ1 leu2Δ0 met15Δ0 ura3Δ0* ***erg2Δ***::*KANMX*	Open Biosystems

YKB4875	*MATa his3Δ1 leu2Δ0 met15Δ0 ura3Δ0* ***erg3Δ****::KANMX*	Open Biosystems
YKB4877	*MATa his3Δ1 leu2Δ0 met15Δ0 ura3Δ0* ***erg5Δ****::KANMX*	Open Biosystems
YKB4876	*MATa his3Δ1 leu2Δ0 met15Δ0 ura3Δ0* ***erg4Δ****::KANMX*	Open Biosystems
YKB5079	*MATa his3Δ1 leu2Δ0 met15Δ0 ura3Δ0* ***ERG10-GFP****::HISMX*	(Ghaemmaghami et al., 2003)
YKB4947	*MATa his3Δ1 leu2Δ0 met15Δ0 ura3Δ0* ***HMG1-GFP****::HISMX*	(Ghaemmaghami et al., 2003)
YKB4948	*MATa his3Δ1 leu2Δ0 met15Δ0 ura3Δ0* ***HMG2-GFP****::HISMX*	(Ghaemmaghami et al., 2003)
YKB5080	*MATa his3Δ1 leu2Δ0 met15Δ0 ura3Δ0* ***ERG12-GFP****::HISMX*	(Ghaemmaghami et al., 2003)
YKB4950	*MATa his3Δ1 leu2Δ0 met15Δ0 ura3Δ0* ***ERG8-GFP****::HISMX*	(Ghaemmaghami et al., 2003)
YKB5081	*MATa his3Δ1 leu2Δ0 met15Δ0 ura3Δ0* ***IDI1-GFP****::HISMX*	(Ghaemmaghami et al., 2003)
YKB5082	*MATa his3Δ1 leu2Δ0 met15Δ0 ura3Δ0* ***ERG9-GFP****::HISMX*	(Ghaemmaghami et al., 2003)
YKB5098	*MATa his3Δ1 leu2Δ0 met15Δ0 ura3Δ0* ***ERG19-GFP****::HISMX*	(Ghaemmaghami et al., 2003)
YKB4949	*MATa his3Δ1 leu2Δ0 met15Δ0 ura3Δ0* ***ERG1-GFP****::HISMX*	(Ghaemmaghami et al., 2003)
YKB5083	*MATa his3Δ1 leu2Δ0 met15Δ0 ura3Δ0* ***ERG7-GFP****::HISMX*	(Ghaemmaghami et al., 2003)
YKB5084	*MATa his3Δ1 leu2Δ0 met15Δ0 ura3Δ0* ***ERG11-GFP****::HISMX*	(Ghaemmaghami et al., 2003)
YKB4951	*MATa his3Δ1 leu2Δ0 met15Δ0 ura3Δ0* ***ERG24-GFP****::HISMX*	(Ghaemmaghami et al., 2003)
YKB5113	*MATa his3Δ1 leu2Δ0 met15Δ0 ura3Δ0* ***ERG25-GFP****::HISMX*	(Ghaemmaghami et al., 2003)
YKB5085	*MATa his3Δ1 leu2Δ0 met15Δ0 ura3Δ0* ***ERG26-GFP****::HISMX*	(Ghaemmaghami et al., 2003)
YKB5086	*MATa his3Δ1 leu2Δ0 met15Δ0 ura3Δ0* ***ERG27-GFP****::HISMX*	(Ghaemmaghami et al., 2003)
YKB4952	*MATa his3Δ1 leu2Δ0 met15Δ0 ura3Δ0* ***ERG6-GFP****::HISMX*	(Ghaemmaghami et al., 2003)
YKB5087	*MATa his3Δ1 leu2Δ0 met15Δ0 ura3Δ0* ***ERG3-GFP****::HISMX*	(Ghaemmaghami et al., 2003)
YKB5088	*MATa his3Δ1 leu2Δ0 met15Δ0 ura3Δ0* ***ERG4-GFP****::HISMX*	(Ghaemmaghami et al., 2003)
YKB5089	*MATa his3Δ1 leu2Δ0 met15Δ0 ura3Δ0* ***ERG5-GFP****::HISMX*	(Ghaemmaghami et al., 2003)
YKB5091	*MATa his3Δ1 leu2Δ0 met15Δ0 ura3Δ0* ***ERG4-GFP****::HISMX* ***CIT1-RFP*** *::URA*	This study

YKB4383	*MATa his3Δ1 leu2Δ0 met15Δ0 ura3Δ0* ***pdr12Δ****::KANMX*	Open Biosystems
YKB5036	*MATa his3Δ1 leu2Δ0 met15Δ0 ura3Δ0* ***erg4Δ****::KANMX* ***pdr12Δ****::NATMX*	This study
YKB4889	*MATa his3Δ1 leu2Δ0 met15Δ0 ura3Δ0* ***PDR12-GFP****::HISMX*	(Ghaemmaghami et al., 2003)
YKB4954	*MATa his3Δ1 leu2Δ0 met15Δ0 ura3Δ0* ***erg2Δ*** *::KANMX* ***PDR12-GFP****::HISMX*	This study
YKB4955	*MATa his3Δ1 leu2Δ0 met15Δ0 ura3Δ0* ***erg4Δ*** *::KANMX* ***PDR12-GFP****::HISMX*	This study
YKB5097	*MATahis3Δ1leu2Δ0met15Δ0ura3Δ0****Can1-GFP****::HISMX*	(Ghaemmaghami et al., 2003)

Transparent Methods

Strains and plasmids

The yeast strains used in this study are listed in Table S2. These yeast strains are derivatives of the haploid BY4741 strain. Strains with GFP tagged proteins were obtained from the GFP collection (Ghaemmaghami et al., 2003) and deletion mutants were obtained from the deletion mutant array collection (Open Biosystems). Strains that were in neither collections were generated using established methods (Longtine et al., 1998). All strains used in this study were PCR confirmed.

Chemogenomic screen and analysis

The chemogenomic screen was performed as previously described (Fletcher et al., 2019)but with a few modifications. Briefly, MATa yeast deletion mutant array (DMA) collection (~4200 mutants) was arrayed in duplicate and condensed at density of 1536 colonies per plate on YPD agar (1% w/v Yeast Extract, 2% w/v Bacto peptone, 2% w/v agar, 0.03% w/v Tryptophan and 2% w/v Glucose) plates supplemented with G418 using a Singer RoToR HDA (Singer Instruments). The condensed arrays were pinned onto YPD agar plates containing 80 mM adipic acid (Sigma-Aldrich; cat. #09582-250G) dissolved in DMSO. The resulting pH of the plates due to the adipic acid was 4.0. A control screen was performed at the same time where the condensed arrays were pinned onto YPD agar plates containing DMSO and the pH of the plates buffered at pH 4.0. Both the adipic acid and the control screens were performed in triplicate and the plates were incubated at 30 °C for 48 h after which images of each plate were taken for growth assessment of each colony using the SGAtools (http://sgatools.ccbr.utoronto.ca/) as previously described (Wagih et al., 2013). An average growth score of less than −0.3 and greater than 0.3 was used as

the cut-off to identify mutants that were sensitive and resistant, respectively, to adipic acid.

Agar-based chemogenomic screen was confirmed by measuring the growth rate of the identified mutants in liquid YPD medium supplemented with 120 mM adipic acid. Briefly, overnight cultures of the mutant strains were inoculated into fresh YPD medium and strains were incubated at 30 °C until they reached the mid log phase (OD_{600} 0.5–0.6), prior to being diluted to a final OD_{600} of 0.1 in a BioScreen C™ Honeycomb microplate. The growth assays were performed for each mutant in triplicate in YPD control media or YPD media supplemented with 120 mM adipic acid. Using a BioScreen C™ plate reader, the cultures were incubated at 30 °C for 72 h and OD_{600} readings were taken every 15 min to plot growth curves. Data from the BioScreen C™ were analyzed with the PRECOG software (Fernandez-Ricaud et al., 2016) to obtain the doubling time of all the strains. A PRECOG score for each of the strains tested was calculated as the ratio of the doubling time in the presence of adipic acid to the doubling time in the absence of adipic acid. Using a cut-off PRECOG score of > 2 and < 0.8 we identified strains that were sensitive or tolerant, respectively, to the adipic acid. The network plot was performed using the statnet package in the R programming software (Handcock et al., 2008).

Gene Ontology (GO) enrichment analysis was performed using the web-based tool (Robinson et al., 2002). The *p*-value cutoff of 0.01 was used to determine clusters that were enriched.

Dot assay and growth rate measurements

Starting with overnight cultures, the wild type and deletion mutant strains were used to inoculate fresh YPD medium to a final OD_{600} of 0.1. The diluted cultures were grown at 30 °C until they reached the mid log growth phase. The cultures at the mid log phase were diluted to an OD_{600} of 0.1 with fresh YPD and four 10-fold serial dilutions of the cultures were spotted on YPD agar plates supplemented with 80 mM adipic acid. Control plates contained no chemical. The YPD agar plates were incubated for 48 hours at 30 °C after which images of the plates were taken with the ChemiDoc XRS Molecular Imaging system (Biorad).

Fluorescence microscopy

Overnight cultures grown at 30°C were used to inoculate fresh YPD to a final OD_{600} of 0.1 and allowed to reach mid-log phase prior to imaging. To perform live cell imaging, the cells were briefly centrifuged (800xg for 2 min), followed by re-suspending in a minimal volume of SC medium (67% w/v yeast nitrogen base without amino acids, 0.2% w/v amino acid drop out mix, 2% w/v glucose). An aliquot of 5 µl was spotted onto glass slides, covered with a cover slip prior to imaging. Images were acquired using either a Leica DMI 6000 florescent microscope (Leica Microsystems GmbH, Wetzler Germany), equipped with a Sutter DG4 light source (Sutter Instruments, California, USA), Ludl emission filter wheel with Chroma band

pass emission filters (Ludl Electronic Products Ltd., NY, USA) and Hamamatsu Orca AG camera (Hamamatsu Photonics, Herrsching am Ammersee, Germany) or an Axio Observer 7 fluorescent microscope was also used to take some images. Images were acquired at 0.2 μm steps across 6 μm using a 63 × oil-immersion objective with a 1.4 numerical aperture.

Image J software was used to quantify fluorescence signal in cells as described by (Schneider et al., 2012) with a slight modification. Briefly, background fluorescence was subtracted from the images and the total fluorescence of all the cells in the image was measured and divided by the number of cells in the image. The images contained a minimum of 50 cells. Normalization was performed by calculating the ratio of fluorescence per cell of treated cultures divided by that of untreated cultures.

Quantitative western blot analysis

Overnight cultures of cells endogenously expressing Pdr12-GFP or Can1-GFP were diluted in fresh YPD medium to a final OD_{600} of 0.1. These were grown at 30°C until they reached the early log phase (4 hours). Subsequently, the cells were centrifuged and resuspended in YPD with or without 120 mM adipic acid and were further incubated for 1 hour. To obtain whole cell extracts (WCE), cells were harvested by centrifugation at 3000 rpm for 3 minutes (4 °C). The cell pellets were then washed with chilled sterile water and flash frozen in liquid nitrogen before storage at -80°C. Cell lysis was achieved using the Trichloroacetic acid (TCA) protocol (Kao and Osley, 2003). For the quantitative western blot analysis, cell abundance was equalized using OD_{600} prior to TCA lysis. Equal volumes of WCE in SDS loading dye were boiled for 10 min and proteins were separated using a TGX stain-free FastCast acrylamide gel (BioRad cat. # 161–0181). After the gel electrophoresis, the TGX gel was activated using UV (Stain-Free Gel program on the Bio-Rad ChemiDoc XRS Molecular Imaging system) and the proteins were transferred onto a nitrocellulose membrane. The total protein on the blot was imaged using the UV (Stain-Free Blot program on the Bio-Rad ChemiDoc XRS Molecular Imaging system) and the blot was blocked with 5% w/v non-fat milk powder in Tris-buffered saline solution with Tween 20 (TBS-T) for 2 h. After the blocking step, the nitrocellulose blots were incubated overnight with Anti-GFP primary antibody (Sigma-Aldrich # 11814460001) in 5% w/v milk TBS-T after which the nitrocellulose membrane was washed (3 × 10 min) with TBS-T. Next, the nitrocellulose blots were incubated for 1 hour with an HRP conjugated anti-mouse secondary antibody (Bio-Rad) diluted in 5% w/v milk TBS-T and then washed (3x10 min) again with TBS-T. In a final step, the nitrocellulose blot was developed with chemiluminiscence reagents (Biorad) and the protein bands were visualized with a ChemiDoc XRS Molecular Imaging system (Biorad). Protein abundance of Pdr12-GFP, Can1-GFP, and Free GFP in untreated versus adipic acid treated samples was quantified using the Image Lab software. This was calculated as the ratio of the Pdr12-GFP, Can1-GFP, or Free GFP band intensity to the intensity of bands in the entire lane signal using the TGX Stain-Free Fast Cast (total proteins). The amount of Pdr12-GFP, Can1-GFP, or Free GFP in the adipic acid treated cultures was then normalized to that of the untreated cultures.

Cellular staining

Starting with overnight cultures, the wild type and deletion mutant strains were used to inoculate fresh YPD medium to a final OD_{600} of 0.1. The diluted cultures were grown at 30°C until they reached the mid log growth phase. The cultures at the mid log phase were stained with the filipin dye (Sigma-Aldrich) for the visualization of sterols in the plasma membrane. To visualize the vacuolar membrane and lumen the FM4-64FX (Invitrogen) and CMAC dyes were used respectively to stain the cells. For the CMAC experiments, the midlog phase cells were stained with the dye at a final concentration of 25 μM for 15 minutes after which the cells were treated with adipic acid. For the FM4-64 experiments, the dye was added at a final concentration of 0.04 mM at the same time the midlog phase cells were grown with or without adipic acid. The stained cells were incubated for 1-2 hours prior to visualization under a fluorescence microscope.

Statistics

Student t-tests and standard deviations were calculated for at least three experimental replicates using the GraphPad Prism 6.05 software (GraphPad Software Inc., La Jolla, California).

Supplemental References

Fernandez-Ricaud, L., Kourtchenko, O., Zackrisson, M., Warringer, J., and Blomberg, A. (2016). PRECOG: a tool for automated extraction and visualization of fitness components in microbial growth phenomics. BMC Bioinformatics *17*, 249.

Fletcher, E., Gao, K., Mercurio, K., Ali, M., and Baetz, K. (2019). Yeast chemogenomic screen identifies distinct metabolic pathways required to tolerate exposure to phenolic fermentation inhibitors ferulic acid, 4-hydroxybenzoic acid and coniferyl aldehyde. Metabolic Engineering *52*, 98-109.

Ghaemmaghami, S., Huh, W.-K., Bower, K., Howson, R.W., Belle, A., Dephoure, N., O'Shea, E.K., and Weissman, J.S. (2003). Global analysis of protein expression in yeast. Nature *425*, 737-741.

Handcock, M.S., Hunter, D.R., Butts, C.T., Goodreau, S.M., and Morris, M. (2008). statnet: Software Tools for the Representation, Visualization, Analysis and Simulation of Network Data. Journal of statistical software *24*, 1548-7660.

Kao, C.-F., and Osley, M.A. (2003). In vivo assays to study histone ubiquitylation. Methods *31*, 59-66.
Longtine, M.S., McKenzie Iii, A., Demarini, D.J., Shah, N.G., Wach, A., Brachat, A., Philippsen, P., and Pringle, J.R. (1998). Additional modules for versatile and

economical PCR-based gene deletion and modification in Saccharomyces cerevisiae. Yeast *14*, 953-961.

Robinson, M.D., Grigull, J., Mohammad, N., and Hughes, T.R. (2002). FunSpec: a web-based cluster interpreter for yeast. BMC Bioinformatics *3*, 35.

Schneider, C.A., Rasband, W.S., and Eliceiri, K.W. (2012). NIH Image to ImageJ: 25 years of image analysis. Nature Methods *9*, 671-675.

Wagih, O., Usaj, M., Baryshnikova, A., VanderSluis, B., Kuzmin, E., Costanzo, M., Myers, C.L., Andrews, B.J., Boone, C.M., and Parts, L. (2013). SGAtools: one-stop analysis and visualization of array-based genetic interaction screens. Nucleic acids research *41*, W591-W596.
